# WatchGuardian: Enabling User-Defined Personalized Just-in-Time Intervention on Smartwatch

**DOI:** 10.1145/3788689

**Published:** 2026-10-26

**Authors:** Ying Lei, Yancheng Cao, Will Ke Wang, Yuanzhe Dong, Changchang Yin, Weidan Cao, Ping Zhang, Jingzhen Yang, Bingsheng Yao, Yifan Peng, Chunhua Weng, Randy Auerbach, Lena Mamykina, Dakuo Wang, Yuntao Wang, Xuhai Xu

**Affiliations:** Simon Fraser University, Surrey, British Columbia, Canada; Columbia University, New York, New York, USA; Columbia University, New York, New York, USA; Stanford University, Stanford, California, USA; The Ohio State University, Columbus, Ohio, USA; The Ohio State University, Columbus, Ohio, USA; The Ohio State University, Columbus, Ohio, USA; Nationwide Children’s Hospital, Columbus, Ohio, USA; Northeastern University, Boston, Massachusetts, USA; Weill Cornell Medicine, New York, New York, USA; Columbia University, New York, New York, USA; Columbia University, New York, New York, USA; Columbia University, New York, New York, USA; Northeastern University, Boston, Massachusetts, USA; University of Washington, Seattle, Washington, USA; Columbia University, New York, New York, USA

**Keywords:** Few-shot learning, Just-in-time intervention, Personalized intervention

## Abstract

While just-in-time interventions (JITIs) have effectively targeted common health behaviors, individuals often have unique needs to intervene in personal undesirable actions that can negatively affect physical, mental, and social well-being. We present WatchGuardian, a smartwatch-based JITI system that empowers users to define custom interventions for personal actions with few samples. To detect new actions from limited data, we developed a few-shot learning pipeline that fine-tuned a pre-trained inertial measurement unit (IMU) model on public hand-gesture datasets. We then designed a data augmentation and synthesis process to train additional classification layers for customization. Our offline evaluation with 26 participants showed that with three, five, and ten examples, our approach achieved an accuracy of 76.8%, 84.7%, and 87.7%, and an F1 score of 74.8%, 84.2%, and 87.3%. We then conducted a 4-hour intervention study to compare WatchGuardian against a rule-based intervention. Our results demonstrated that our system led to a significant reduction by 64.0 ± 22.6% in undesirable actions, substantially outperforming the baseline by 29.0%. Our findings underscore the effectiveness of a customizable, AI-driven JITI system for individuals in need of behavioral intervention in personal undesirable actions. We envision that our work can inspire broader applications of user-defined personalized intervention with advanced AI solutions.

## 1 Introduction

Recent advances in mobile sensing technologies and AI have led to the emergence of research on intelligent, **just-in-time interventions (JITIs)** using mobile or wearable devices [3, 72, 77, 78, 114, 122, 127, 172, 173]. A typical research paradigm usually starts by identifying a target undesirable behavior, followed by data collection from mobile and/or wearable devices, **machine learning (ML)** model development, and finally, real-time system evaluation (e.g., [3, 38, 62, 72, 104, 140, 165, 166, 171]). When deployed, these systems detect the occurrence of target behaviors and deliver JITIs to help users regulate their behaviors and achieve personal health goals. In the past decade, researchers have achieved a wide range of successful JITI applications, such as reducing smartphone overuse [83, 163], prevention of sedentary habits [78], smoking cessation [3], promoting skin health [72, 122, 140], and managing stress and emotions [38, 62, 65].

Existing research predominantly focuses on *common* health behaviors that are generally applicable to a large group of populations. However, some individuals’ undesirable behaviors can be highly *personal* and *idiosyncratic*. This is especially the case for personal micro-actions or micro-habits. Example actions include leg-shaking, nail-biting, hair-pulling, and skin-picking (some referred to as **body-focused repetitive behaviors (BFRBs)**) [105, 110, 128, 135–137, 143, 151]. Such micro-actions can have negative impacts on ones’ health (e.g., lip-picking can cause cheilitis symptoms [14, 35]), or unfavorable social implications (e.g., leg-shaking is considered rude and disrespectful in some cultures [168]). These actions vary considerably across individuals [161], shaped by diverse physical, psychological, social, and environmental factors [143]. Consequently, developing a personalized JITI system poses substantial challenges in both data collection and model training. From a data-collection perspective, it is impractical to require users to gather extensive real-world samples of every undesirable action. From a modeling perspective, training a robust system on such limited data to provide personalized interventions that are adapted to individual contexts and goals remains difficult.

To address this gap, we built *WatchGuardian*, a smartwatch-based system enabling users to go beyond pre-defined undesirable actions and easily customize new interventions targeted at their own specific undesirable actions (Figure 1). We developed a few-shot learning pipeline that only requires a small number of samples of the individual target behavior and outputs a reliable ML model for customized behavior detection and real-time JITIs. Specifically, we built on top of a pre-trained **inertial measurement unit (IMU)** model based on **self-supervised learning (SSL)** [169] and fine-tuned the model on multiple open IMU datasets of hand-gesture recognition, with the goal to enhance the model’s feature embedding capability on fine-grained actions. Then, given the small sample sizes of new target behaviors, we adopted a series of data augmentation and data synthesis techniques to train additional lightweight classification layers for the new custom undesirable actions for each individual.

We evaluated our system through both an offline evaluation experiment and a real-time intervention study. For the offline evaluation, we pre-determined a set of five micro-actions that are typically considered negative behaviors and can be captured with a wrist-worn smartwatch, including face-scratching, nail-biting, eye-rubbing, lip-picking, and leg-shaking [105, 135–137, 143]. We then collected data from participants (N = 26) on these five actions. We also ask participants to self-define new wrist-based actions that they want to receive intervention. Our final model achieves an average accuracy of 76.8%, 84.7%, and 87.7%, and an F1 score of 74.8%, 84.2%, and 87.3% with one, five, and ten examples. Building on the model, to evaluate the intervention effectiveness of WatchGuardian, we conducted another 4-hour-long study (N = 21) that simulated real-life intervention experience. We compared our system against a rule-based intervention method in an environment where participants naturally tended to perform their self-chosen actions. The results indicate that WatchGuardian reduced undesirable actions by 64.0 ± 22.6% with statistical significance (p < 0.05), and our system substantially outperformed the baseline intervention method by 29.0% (p < 0.05). To test its real-world applicability, we further conducted a proof-of-concept study in the wild (N = 3), with each participant used the system for 3 days. Participants’ qualitative feedback also revealed interesting insights into the human–AI intervention experience, including participants’ distorted perceptions of the intervention’s strength and effectiveness, and various collaborative relationships between users and AI. The effectiveness of WatchGuardian to mitigate personal undesirable behaviors, shown by both an offline evaluation experiment and a real-time intervention study, sheds light on the future design of personalized AI-powered JITI systems. Overall, our contributions can be summarized as:

- We introduced WatchGuardian, the first smartwatch-based JITI system that empowers users to define personalized intervention on undesirable micro-actions.
- We conducted an offline evaluation of our few-shot learning pipeline by recognizing different numbers of undesirable actions and numbers of few-shot samples. This extensive evaluation indicates the robust performance of our pipeline.
- We implemented WatchGuardian as a real-time intervention system and conducted a user study to evaluate its effectiveness. Our results not only show its advantage over the baseline, but also reveal a range of interesting insights that can guide the future design of human–AI intervention systems.

## 2 Related Work

In this section, we first provide a general overview of just-in-time behavior intervention, and then a review of prior work in hand gesture recognition based on wearable devices.

### 2.1 Sensing-Based JITI

Advances in mobile sensing technologies enable the unobtrusive real-time monitoring of individual states and environmental contexts, while delivering proactive cues and user-specific information [20]. Such advances facilitate the implementation of JITI [99], with the goal of delivering timely and appropriate support for users. Early research has applied JITI to address a variety of health-related issues using rule-based approaches [20, 37, 43, 44, 59, 60, 85, 115, 141]. These approaches usually depended on pre-defined sets of rules and conditions to trigger interventions, which are typically defined by domain experts. Examples include event-based rules [59, 60, 115], time-based rules [37, 85, 141], combinations of multiple rules [43], and multi-stage rules [20], to name a few.

Recently, with the advancement of AI techniques, an increasing number of studies have started to apply AI for JITI [3, 62, 72, 77, 78, 104, 127, 157, 163]. In contrast to rule-based JITI, AI-based approaches utilize large-scale user behavior data and trained AI/ML models to determine optimal intervention timing and personalized interventions. For instance, Time2Stop [104] employs ML to develop an adaptive, explainable intervention system for smartphone overuse that determines optimal timings, offers transparent AI explanations, and integrates user feedback to improve the model over time. Liao et al. [78] and Rabbi et al. [114] incorporated reinforcement learning algorithms into JITI systems to personalize the model for each user, enhancing the effectiveness of physical activity interventions.

However, these studies primarily focus on pre-defined *common* health behaviors that are broadly applicable to large populations, failing to address personalized and idiosyncratic undesirable behaviors that are unique to individual users [105, 135–137, 143]. Although there are some research focusing on applying JITIs on some BFRBs [128], such as nail-biting [110], scratching [72], and face-touching [122, 140], they still only work for specific and relative common BFRBs and cannot support broader idiosyncratic undesirable behaviors. To support JITIs in personalized idiosyncratic behaviors, an intelligent intervention system needs to address the challenges that the sample size (from a single individual) would be much smaller than common health behaviors. The limitation of existing solutions reduces the system’s ability to adapt to personalized behaviors. To bridge this gap, our work proposes a personalized intervention approach to deliver customized JITI for user-defined undesirable actions.

### 2.2 Wearables for Hand Gesture Recognition and Customization

The field of hand gesture recognition or activity recognition using wearable technologies has been extensively studied, utilizing a range of sensing techniques (e.g., vision [33, 45, 58, 102, 113, 156, 158, 167], sound wave [40, 51, 52, 69, 71, 76, 100], electromyography [15, 92, 117, 118, 125, 126], pressure or stretch [24, 25, 55, 132, 139], and magnetism [11, 18, 19, 57, 107, 134, 164]). Among them, motion data (e.g., acceleration, angular velocity) collected by IMUs are particularly notable for their effectiveness in capturing dynamic hand gestures [1, 61, 70, 75, 129, 154, 159]. Coupled with their cost-effectiveness and widespread availability in commercial wearable devices, IMUs’ are the best choice of sensor for a JITI system to ensure effectiveness, ubiquity and generalizability.

Existing gesture recognition approaches can be broadly categorized into trajectory-based and ML-based. Early trajectory-based methods [79, 89] achieve high accuracy with relatively few samples, but they are limited in recognizing more complex gesture trajectories [89]. For more sophisticated and fine-grained gestures, ML-based methods are more suitable, but are typically heavily data-driven, requiring a large number of samples of a pre-defined gesture set to train either a traditional model [16, 32, 46, 51] or a deep learning model [10, 46, 54, 73, 75, 128–130] depending on the dataset size.

In addition to recognizing gestures from pre-defined sets, some systems support the customization of user-defined gestures, which enhances memorability [97], interaction efficiency [106], and accessibility for individuals with physical disabilities [4]. Most notably, incorporating gesture customization in our system enables users to define their own undesirable gestures for intervention. Such user-driven training, adapting systems based on user-provided data, is common not only in gesture recognition [146, 160], but also across a wide range of mobile and wearable systems, such as routinely tracking screen usage [104], stress level [38], and physical activity [78], to improve algorithm and adapt interface content or layouts to individual users.

However, to ensure a seamless user experience in gesture customization, the data collection process for new gestures must be efficient and limited in scale (e.g., no more than 10 samples). Existing approaches (e.g., rule-based methods [6, 30] and computational techniques [5, 82, 89, 106]) meet this requirement, but are limited to recognizing hand gestures with significant motion, where IMU signals are distinct, and the recognition task is relatively straightforward. To identify more sophisticated and fine-grained gestures or actions, there are two common approaches: (1) collect more data of the new gesture to further train models, or (2) fine-tune a pre-trained base model use a limited amount of new samples. Since the first option would significantly impact user experience, the fine-tuning approach is more appropriate, particularly when combined with human knowledge and lightweight model adaptation strategies that balance human input and automated learning [174]. However, fine-tuning a robust fine-grained gesture recognition model with a small number of samples remains a challenging task [54, 116–118, 138, 155, 161, 175]. Most related to our work, Xu et al. [161] collected extensive gesture data to pre-train a robust model and used few-shot samples to fine-tune new gestures, but this work focuses on short-duration gestures (less than 1 second), which may limit its potential to support users in defining their own undesirable actions for intervention. Furthermore, the work is closed-sourced, with both its dataset and pre-trained model unavailable for public use, hindering reproducibility and broader applicability.

Building on top of prior work to address the challenges of gesture customization, we developed an open-sourced few-shot learning pipeline using public models and datasets. Our system enables the model to quickly adapt to new target gestures or actions with high accuracy using only a few samples.

## 3 WatchGuardian Design

We designed a few-shot learning pipeline to enable users to define their own undesirable actions with a small number of examples. We introduce our technical pipeline (Section 3.1), the interface and intervention experience design (Section 3.2), as well as the implementation details of WatchGuardian (Section 3.3).

### 3.1 Few-Shot Learning Pipeline

To achieve the goal of learning user-defined undesirable actions with few-shot samples, we designed and implemented a three-stage pipeline building on public models and datasets. Figure 2 visualizes the overall structure of the pipeline. To ensure compatibility with existing public models and datasets, we used tri-axial accelerometer data from the IMU, sampled at 30 Hz.

#### 3.1.1. Stage 1: Pre-Trained Model.

One of the primary challenges in deep learning for **human activity recognition (HAR)** is the lack of large labeled datasets [73, 169]. Although there exist multiple public HAR datasets with ground truth labels, they often have limited size and different sensing modalities, data sizes, task definitions, and collection protocols (e.g., [2, 16, 23, 42, 46–47, 64, 68, 87, 88, 103, 121, 148]). Therefore, it is challenging to unify these datasets into a single large-scale dataset for pre-training a supervised learning-based model. SSL addresses these challenges by leveraging vast amounts of unlabeled data to learn meaningful representations through pretext tasks, making it particularly well-suited for HAR tasks [123].

We adopted a pre-trained model developed by Yuan et al. [169], which was trained using multi-task SSL on the UK Biobank activity tracker dataset [27]. The dataset contains over 700,000 person-days of unlabeled wearable sensor data collected from free-living activities via a wrist-worn accelerometer. This dataset setup fits well into our target application scenarios. Figure 2(a) provides a high-level overview of the architecture of the pre-trained model, which includes a five-layer ResNet-based feature extractor [41] followed by two fully connected layers. The model was pre-trained on three fixed-length signals (i.e., 5 seconds, 10 seconds, and 30 seconds) at 30 Hz. Three data augmentation techniques [147], including Arrow of Time, Permutation, and Time Warping, were used in pre-training process as three self-supervised tasks [123]. This pre-trained model is publicly available.^1^

#### 3.1.2 Stage 2: Model Fine-Tuning.

While direct feature extraction from IMU signals using the pre-trained model has demonstrated improved performance in downstream classification tasks [169], there is a significant gap between the pre-trained tasks (i.e., mostly coarse-grained human activities that involves large range of motion) and our target customization tasks (i.e., actions with fine-grained activity). This brings up the need for better model adaptation to bridge this discrepancy. To address this gap, we fine-tuned the pre-trained SSL model using two datasets curated by Hu et al. [46] and Bhattacharya et al. [10] that are closer to our use cases in a supervised manner.^2^ These datasets contain labeled, fine-grained, hand-specific human activity data.

We pre-processed the datasets before merging them together. We first unified their units, resampled them to 30 Hz to maintain uniformity across datasets, and then applied z-score normalization to standardize the signal. After these steps, we adopted a 5-second sliding window with a step size of 0.1 second, because a 5-second window fits one of the pre-training signal lengths in the SSL model in Section 3.1.1 and is appropriate for our use cases (the same window size as described in Section 3.1.3). As for the labels, we manually unified activities with the same semantics (e.g., handwriting in [48] and writing in [10]), resulting in a combined dataset with 26 activity classes (defined as *positive classes*) plus one class for the rest without target activity (defined as *negative class*).

To establish a more comprehensive and diverse representation of the negative class, we recruited a small group of participants (N = 10), each performing 30 minutes of regular indoor daily activities, such as sleeping, playing computer games, studying, and cooking. Participants were specifically instructed and supervised to minimize potentially undesirable micro-actions to ensure the quality of negative data. We manually remove the improper data episodes from the data. This data was then sampled for the subsequence training process. This step enables the model to learn from more comprehensive and diverse negative class samples, thereby reducing false positives.

Combining all these datasets together resulted in a dataset with about 12.5 hours of signals of positive classes and 5 hours of signals of negative classes (before sampling) in total. Using this combined dataset, we performed fine-tuning on the pre-trained model, with all layers activated (see Figure 2(b)). We adopted weighted cross-entropy as the loss function to address the class imbalance problem.

#### 3.1.3 Stage 3: Few-Shot Model Customization.

As mentioned earlier, in real-world applications, it is often impractical for users to provide a large number of samples of a self-defined action for model training. To achieve individual customization, our few-shot learning procedure was designed to train a new prediction head with lightweight layers built upon the fine-tuned model from Section 3.1.2. For easy understanding, we will explain our pipeline with the case of adding one intervention action (i.e., binary classification) in detail, starting with data collection and then the few-shot learning process. The scenarios with multiple actions adopt the same method.

We implemented a simple, user-friendly data collection process for customization, where a user would follow instructions on the wearable device to repeat the target action several times (N shots, 10 seconds each time), with a short period of 5-second pause or other activities (negative class) between the two repetitions, as shown in Figure 2(c). Given such a signal sequence, we applied the same sliding window process as in Section 3.1.2 (5-second width and 0.1-second step). The sliding window width was chosen to ensure each window captures a full instance of the target undesirable action (e.g., BFRBs). These actions typically last longer and repeat over time, distinguishing them from brief gestures or incidental movements. The step size follows prior work in wearable human action recognition, especially with limited data in few-shot learning settings [161]. Each window was labeled as positive if it contained more than 3 seconds of the target action; otherwise, it was annotated as negative.

We then introduced a signal processing procedure, including both data augmentation and data synthesis, to enrich the training data and improve the model’s ability to generalize to gestures that do not exactly match the originally collected samples. First, we adopted six data augmentation techniques [48]: (1) zooming, to simulate variations in action speed, randomly selected from ×0.9 to ×1; (2) scaling, to represent variations in action intensity, with the scaling factor *s* ~ 𝒩(1, 0.2^2^), *s* ∈ [0, 2]; (3) time warping, to simulate action temporal variance, using two interpolation knots and a warping randomness *w* ~ 𝒩(1, 0.05^2^), *w* ∈ [0, 2]; (4) time reversal, to simulate temporal variation, by reversing the action’s time sequence; (5) time-domain noise, to simulate sensor inaccuracies or environmental disturbances, Gaussian noise with a noise level of 0.01 is added to the original data; and (6) frequency-domain noise, to simulate frequency variation or external interferences. We add the random noise to the frequency components of the signal (after Fourier transform) and then transformed the signal back to the time domain (with inverse Fourier transform). We went through all combinations of these six augmentation technique steps, which increased the size of the data by 2^6^ − 1 times.

Next, we designed a data synthesis step. To create additional samples that simulate short episodes of target undesirable actions, we artificially concatenated short segments of the target undesirable action with negative episodes (no target undesirable actions). The positive segments (of the target undesirable actions) were chosen randomly from the 10-second continuous recordings and would have varying lengths sampled from [3, 4.9] seconds. The starting position of the positive episode was also randomized within a 5-second window, with negative episodes padding at the beginning and the end. This step further increased the sample size by about 80 times. In total, our data augmentation and data synthesis steps enlarged the original few-shot samples by about 143 times. Although the dataset was substantially expanded, all augmented and synthesized samples were generated as controlled transformations or combinations of the user’s original IMU signals, ensuring that the expanded dataset remained distributionally close to real user behavior.

Finally, to customize the model to recognize a new target undesirable action, we further trained the fine-tuned model with a new classification head with the total set of data. The head was trained for a binary classification when adding one intervention action, and N + 1-class classification when adding N new actions.

In the real-time system, the final classification model followed the same sliding window setup and performed classification at 10 Hz (0.1-second step). To improve the system robustness, we added a smoothing step with the threshold as 3 based on grid search, i.e., the system will only recognize a target action if there is a consecutive sequence of positive outcomes from three windows. This helps reduce false detections when facing transient fluctuations or noise.

### 3.2 Intervention Design

Building upon the customized model, we then developed a real-time intervention system on the smartwatch. As introduced in Section 3.1.3, in the initial customization process, a user would go through a simple data recording to collect a few samples of the undesirable action. Figure 3(a) shows the interface on the smartwatch to select or define an undesirable action and set up the number of shots for customization.

Once a model was trained following the 3-stage pipeline, the user could enter the live-stream mode to receive interventions. Whenever the target action was detected, the watch would send a reminder notification with vibration. Figure 3(b) shows the interface of the intervention. The user could click a button to dismiss the reminder. To avoid delivering overwhelming interventions, we set a 5-minute cool-down time, i.e., at most, one intervention can be delivered during this interval.

### 3.3 System Implementation

We adopted a client-server architecture for the system implementation to enable efficient data transmission and processing. The interface was implemented on the Google Pixel Watch 2, which acted as the client device. It continuously streamed the accelerometer data, collected at 30 Hz, to a dedicated server in real time via a socket communication protocol.^3^ Before real-time data transmission, the server had already completed the initial setup stages, namely Stage 1 and Stage 2, as described in Section 3.1. Therefore, once the customization data from the client was collected, we utilized an A100 GPU to perform the few-shot custom model training, enabling rapid adaptation to new data with minimal samples. After training the model on the server, we deployed the final model for real-time inference. The inference process ran on the server, and the results were transmitted back to the client for immediate feedback, enabling efficient and responsive action recognition and the delivery of JITI.

## 4 Model Evaluation

In this section, we report the evaluation of WatchGuardian’s few-shot learning pipeline offline performance. We will further elaborate on the evaluation of WatchGuardian’s intervention effectiveness in Section 5.

### 4.1 Data Collection

#### 4.1.1 Participants.

We recruited 26 users (14 females, 12 males, age 22 ± 2) for data collection via social media platforms. We focused on users who were aware of their own undesirable actions and had the intention to reduce these actions. These are the target users of our intervention system. Our study was IRB-approved by the local institution, and participants were compensated with *$*10 for this data collection study (around 45 minutes).

#### 4.1.2. Personal Undesirable Action Customization.

Participants were asked to record five pre-determined target actions that are commonly recognized as undesirable actions [105, 143], including *Face Scratching*, *Nail Biting*, *Eye Rubbing*, *Lip Tearing*, and *Leg Shaking*. The first five figures in Figure 4 illustrate these actions.

Moreover, each participant was asked to define a new undesirable action tailored to their own personal needs. In total, 26 participants designed an additional set of 12 actions, including *Finger Lipping* (designed by N = 5 participants), *Head Scratching* (N = 5), *Nose Rubbing* (N = 4), *Finger Picking* (N = 3), *Hair Scratching* (N = 2), *Face Rubbing* (N = 1), *Finger Biting* (N = 1), *Hair Pulling* (N = 1), *Hair Rubbing* (N = 1), *Lip Biting* (N = 1), *Nail Picking* (N = 1), and *Neck Scar Scratching* (N = 1). We only grouped identical actions and distinguished actions as long as they differed slightly. For instance, *Head Scratching* and *Hair Scratching* were similar, but one involved contacts between fingers and scalp, while the other one did not. Similarly, *Finger Picking* and *Nail Picking* were also quite close, yet one solely focused on the skin on the finger, while the other focused on nails. These actions were visualized in the second half of Figure 4.

#### 4.1.3 Data Collection Procedure.

For each action, participants followed a consistent protocol (briefly mentioned in Section 3.1.3) comprising two phases per shot: a 5-second *free mode* and a 10-second *record mode*. In the free mode, participants were free to rest or perform natural daily activities (negative data). Once entering the record mode, they performed the target actions (positive data). This process was repeated across five rounds, with each round consisting of five consecutive shots. Participants took a short break between two rounds to prevent physical fatigue and were asked to freely adjust the watch position between each round to increase data variance. In total, we collected 25 shots for each target action. Moreover, we leveraged the onboarding process at the beginning of the data collection to passively record participants’ natural activities (about 5 minutes). This was used as additional data to augment the negative class.^4^

The *free mode* segment was labeled as negative data, while the *record mode* segment was labeled as positive data. To prevent data contamination, the first 2 seconds during the record mode were excluded from training because these recordings were mixed with postural changes and arm movement.

### 4.2 Offline Performance Evaluation

We evaluated our pipeline offline by adding one or more actions as target actions. For each action, we randomly selected two rounds of recordings as the training set (up to 10 shots), one round as the validation set (5 shots), and the remaining two rounds as the test set (10 shots). We repeated the training three times and calculated the average performance.

It is noteworthy that the model performance has two aspects: the window level and the action level. For the window level, each sliding window is counted as a binary classification data point (same as the model training process). For the action level, windows are aggregated with a smoothing threshold of 3 (Section 3.1.3) and represent a closer experience as real-life applications. Such aggregation significantly reduces the false negative and false positive.

#### 4.2.1 Prediction Performance with Different Number of Shots and Actions.

We evaluated the model performance by training on one to ten shots of the data. For action recognition, we started by adding one action for each participant (i.e., training binary classification models). To evaluate the performance of multi-class classification models, we also experimented with customizing multiple actions (up to six, as each participant recorded five pre-designed actions and one custom action). This led to a total number of 63 combinations from one to six actions ($\sum_{k=1}^{6}\left(\begin{array}{c} 6\\ k \end{array}\right)$). In total, we trained and evaluated 49,140 models = 10 shot numbers × 63 action combinations × 26 participants × 3 repetitions.

We mainly focused on the action-level performance. Table 1 presents both the window-level and action-level results. More results of few-shot pipeline performance with different numbers of actions and shots are shown in Tables 1 and 2 in the supplementary material. As shown in Figure 5, when using only one shot to add a new action (i.e., the user performs the action only once), our framework achieved an average accuracy of 76.8% and an F1 score of 74.8%. The recognition performance became better with more shots for training the model. With five shots of a new action, our framework attained an average accuracy of 84.7% and an F1 score of 84.2%. When using 10 shots, our model’s performance achieved 87.7% and 87.3%, respectively.

Recognizing multiple new actions simultaneously presented a greater challenge. However, compared to the performance of adding one action with five shots (84.7% and 84.2%), introducing three new actions (i.e., four-class classification) with five shots each, the framework maintained a good average accuracy of 79.1% and an F1 score of 78.1%. Even with six additional new actions and five shots each, the framework still achieved an average accuracy of 73.7% and an F1 score of 72.3%. These results demonstrated the robustness and effectiveness of our pipeline for data-efficient action recognition.

#### 4.2.2 Prediction Performance of Each New Gesture with Different Number of Shots.

We further compared the recognition performance across actions. As shown in Figure 6, most of the 17 actions exhibited good performance. Using only one shot, about half of the actions achieved an F1 score above 75%. When the number of shots increased to five, 14 out of 17 actions surpassed this threshold. With 10 shots, performance improved further for most actions, with 12 out of 17 actions achieving an F1 score above 85%. *Hair Pulling* appeared to be an exception. Its performance did not improve with more samples after five shots. This was probably due to the overly large variance of the *Hair Pulling* action, even performed by the same individual, and it was challenging for a model to achieve reliable performance even with a limited amount of additional data.

Overall, these results indicate our framework has good learning ability for new actions.

#### 4.2.3 Comparative Evaluation and Ablation of Pipeline Stages.

We compared our methods’ performance against baseline methods. Specifically, we experimented with two traditional ML baselines (SVM and Random Forest) and three task-specific methods—COVID-away [140] and Itchtector [72] for undesirable behavior intervention, and HandGesCus [161] for few-shot hand-gesture customization. In addition, to isolate the impact of each pipeline stage, we further performed an ablation study by sequentially incorporating Stage 1 (pre-trained SSL model) [169], Stage 2 (fine-tuning), and Stage 3 (data augmentation and synthesis). For consistency, the same training and testing data were used across approaches and conditions, focusing on adding one new target action with 5-shot samples of training data. The results of the 10-shot condition are shown in Table 3 in the supplementary material.

Table 2 reports action-level performance. Our three-stage pipeline outperformed both traditional ML baselines and task-specific approaches proposed in prior work, achieving the highest scores across all metrics. Our model showed a relative improvement of approximately 6–20% in F1-score. Our results also demonstrate that classification performance improves with three stages. Notably, the addition of the pre-trained SSL model (Stage 1) accounts for the largest single gain (Δ = 8.3% increase in accuracy), followed by data augmentation and synthesis (Stage 3, Δ = 5.8%).

In addition, we note that ML models (i.e., SVM, Random Forest, and Itchtector) tend to perform relatively well in limited-data regimes compared with WatchGuardian in its early stages. For fair comparison, we applied our data augmentation and synthesis techniques to these baselines. Unlike WatchGuardian, these traditional models did not benefit much from augmented data and even showed degraded performance (e.g., F1-score of Random Forest drops from 0.761 to 0.670). This can be attributed to their stronger inductive bias and reliance on fixed or hand-crafted features, which allow efficient learning from small datasets but limit their capacity to exploit the increased feature diversity and intra-class variability introduced by augmentation [9, 36, 81]. In contrast, our deep learning-based pipeline, with its hierarchical neural network structure and large number of parameters, can learn more complex and expressive feature representations, effectively leveraging the augmented diversity to improve generalization [13, 133, 170].

## 5 Intervention Evaluation

The promising model performance in Section 4.2 has validated the effectiveness of our few-shot learning pipeline. Building upon the pipeline, we further conducted a user study to evaluate the effectiveness of WatchGuardian and compared it against a rule-based baseline intervention system.

### 5.1 Participants

With IRB approval, we recruited the same set of participants in Section 4.1 for a follow-up intervention study. In the previous data collection, participants performed five pre-determined actions and a self-defined action. In this study, they were asked to select one of the six actions that they had the strongest need for intervention. This action was set as the target action for intervention during the study. The model used for intervention was trained using 10-shot data samples. Among the 26 participants, 5 of them did not follow the study protocol. Their results were removed as outliers. This section focuses on the findings based on the remaining 21 participants. This sample size is considered statistically sufficient within the HCI community, consistent with prior studies [39, 63, 120].

### 5.2 Intervention Setting

Since personal undesirable actions are inherently difficult to predict or control, we designed an intervention experience that closely mirrors real-life contexts to enhance ecological validity, encouraging participants to perform these actions under more natural conditions. Our initial conversation with participants indicated two common scenarios where they tended to perform these actions: when they were in an engaging task with a relaxing state (e.g., watching an interesting movie or a reality show with dramatic twists and turns); and when they were bored or disengaged (e.g., mindlessly scrolling through social media or watching a tedious video).^5^ Therefore, we set up two types of video-watching tasks and allowed participants to pick the type in which they tended to perform more undesirable actions.

The first type included *engaging* videos. We prepare a set of multi-hour videos for participants to choose from, such as the Harry Potter movie series, sports competitions, and mystery/detective shows. The second type was watching *disengaging* videos. Examples include cycling or driving route videos, math problem explanations, and public health lecture videos. Participants sat in a quiet room with a laptop on the table and watched the video they selected, as shown in Figure (a) and 7(b). During the video-watching, participants were not interrupted by the experimenter, simulating the real-life setting. Note that all participants were instructed to wear the smartwatch on their dominant wrist, consistent with common settings in smartwatch sensing evaluation studies [, 84].

### 5.3 Study Design and Procedure

We adopted a within-subject design and compared our AI-powered WatchGuardian against a rule-based intervention system. In the rule-based system, a regular notification (the same interface as Figure 3(b)) was delivered every 10 minutes, regardless of whether the user did the action. To mitigate the effect of the two systems outputting different numbers of notifications, we further added restrictions in WatchGuardian so that the number of delivered notifications would be in the range of ×0.5 to ×2 as the baseline system. This was achieved by forcefully delivering a notification if there was no intervention by the end of each 20-minute window (×0.5 times of interventions in minimum). With the 5-min cool-down setup, WatchGuardian can only deliver up to one intervention every 5 minutes, which would be no more than ×2 times of interventions as the baseline.

Our study procedure was designed as follows. After selecting the personal target undesirable action and the task type (engaging vs disengaging), participants would calibrate and familiarize themselves with the intervention system and study setup. They then attended two intervention sessions in total, one session per day. We counterbalanced the order between WatchGuardian and the baseline system, and participants were blind to the order of the two systems. After familiarizing themselves with the room environment and setup, participants went through each intervention session with three stages (in total 130 minutes): (1) a 30-minute *pre-intervention stage*, where there was no intervention delivered; (2) a 90-minute *intervention stage*, where WatchGuardian or the baseline system would deliver interventions as designed; and (3) a 10-minute *post-intervention* stage, where no more intervention was delivered to observe any lasting effect.

The whole intervention session was video-recorded by a camera from the ceiling, positioned at an angle to capture participants’ micro-actions and collect ground truth (see Figure 7(c)). We manually annotated the video and calculated the number and duration of the target actions during the three stages. We collected participants’ Self-Report of Habit Strength of the target action [149] before and after each session. After the post-intervention stage, we further collected quantitative data from participants with a questionnaire that includes **system usability scale (SUS)** survey [8] and **working alliance inventory (WAI)** [96]. In addition, we conducted a brief semi-structured interview to collect qualitative feedback on the intervention experience from each participant.

In total, the two sessions took around 5 hours for each participant, Participants were compensated with $50 for the intervention study.

### 5.4 Proof-of-Concept Longitudinal Study

To assess the long-term user experience of WatchGuardian in a real-world daily context, we recruited three external participants to take the smartwatch home to conduct an additional proof-of-concept longitudinal study to enrich our evaluation results.^6^ They were asked to wear the smartwatch for 3 days during the following times: 9–12 AM, 2–5 PM, and 7–9 PM. The smartwatch was charged during experimental breaks. No specific tasks were assigned during the experimental period, allowing their activities to more closely reflect real-life situations. Participants were asked to use a just-in-time diary to record their feelings and experiences after being notified by WatchGuardian. After the experimental phase, participants were asked to share their diary entries and elaborate on their feelings and experiences with WatchGuardian throughout the 3-day intervention.

### 5.5 Intervention Results

We first summarize the quantitative results from our study. We coded the recorded videos by documenting the duration of target actions performed by participants every 10 minutes across the three stages. Since participants had diverse behavior patterns, we normalized the results with each individual’s target action duration in the pre-intervention stage as the reference. The *relative duration* was calculated by dividing the average duration of target actions per 10 minutes in both the intervention and post-intervention stages by that of the pre-intervention stage. A lower relative duration means more reduction of the target actions compared to the pre-intervention stage.

We also evaluated the performance of the WatchGuardian during the user study.^7^ On average, our system achieved an accuracy of 0.653 and an F1 score of 0.750, recording 1.52 false positives per hour. Although its performance in this real-world setting was slightly lower than in offline evaluations in Section 4, the user experience and the effectiveness remained quite satisfactory, as shown below. Furthermore, both the potential benefits and the impacts of misclassifications are introduced in greater detail in Section 5.6.

#### 5.5.1 Reduction of the Duration of Target Actions by Intervention.

We compare the relative duration between WatchGuardian and the baseline in both intervention and post-intervention stages. Since participants received slightly more notifications in WatchGuardian during the intervention stage (on average 11.8 vs. 9.0 times per session), we controlled the effect of the number of notifications by using **generalized linear mixed models (GLMMs)**. Specifically, a GLMM had relative duration as the dependent variable, with the intervention method (AI-based in WatchGuardian vs. rule-based in baseline) and the number of notifications as the main factors.

As shown in the left of Figure 8(a), during the intervention stage, WatchGuardian resulted in 36.0 ± 22.6% of the duration compared to the pre-intervention stage (i.e., a reduction of 64.0% of the target undesirable action), and the baseline system led to 65.0 ± 47.5% of the duration (i.e., a reduction of 35.0%). We fitted a GLMM to compare the two intervention methods. Our results revealed the significant difference between the two methods: WatchGuardian significantly outperformed the baseline by 29.0% more reduction of the target undesirable action ($\chi_{1}^{2}=6.32$, p < .05). Meanwhile, the number of notifications does not show significance ($\chi_{1}^{2}=0.53$, p = 0.47). These results suggest that the advantage of WatchGuardian was mainly attributed to the AI-based intervention method.

In addition, although our post-intervention stage was short, both methods showed promising signals of a potential lasting effect when the intervention was gone (13.9 ± 16.8% for the WatchGuardian; 37.7 ± 37.2% for the baseline), as shown in the right of Figure 8(a). We fitted another GLMM on the post-intervention data. The results also indicate the significance of the intervention method ($\chi_{1}^{2}=10.04$, p < 0.01), but not the number of notifications ($\chi_{1}^{2}=0.12$, p = 0.73). This is consistent with the result of the intervention stage, further demonstrating the superior performance of WatchGuardian over the baseline method.

#### 5.5.2 Intervention Effectiveness over Time.

To investigate changes in the duration of target action during the study session, we visualize the change of participants’ target action duration throughout the study (see Figure 8(b)). Both intervention methods showed a clear and significant decreasing trend once participants entered the intervention stage. The fitted lines in Figure 8(b) indicate that WatchGuardian achieved more duration reduction (*m* = −4.8% per 10-minute) compared to the baseline (*m* = −4.1%) over the intervention session. In particular, WatchGuardian had a more rapid initial decrease and maintained consistently lower levels throughout the rest of the session compared to the rule-based baseline. Overall, WatchGuardian demonstrated stronger cumulative effects.

#### 5.5.3 Difference across Task Types.

During the study, we asked participants to pick their own preferred task types between watching engaging (N = 11) or disengaging videos (N = 10). Figure 9 presents the breakdown of the task type in Figure 8(a). We fitted GLMMs with task type as another main factor and observed a marginal significance of the interaction between the intervention method and the task type ($\chi_{1}^{2}=3.27$, p = 0.07 < 0.1). This was only during the intervention stage, but not the post-intervention stage. Figure 9(a) and (b) indicates that the advantage of WatchGuardian during the intervention stage was more salient when participants were watching engaging videos (Δ = 42.3 ± 49.6%) compared to when they were watching disengaging videos (Δ = 14.2 ± 22.5%). This could be due to the fact that participants were more interruptable or receptive in less engaging tasks [95, 108], thus even a basic rule-based intervention could effectively reduce the target actions. However, in more engaging tasks, accurate and just-in-time reminders are more effective than basic ones.

#### 5.5.4 Survey Outcomes.

In addition to the objective measurement, we also compare participants’ subjective reports on the SUS, WAI, and the change of the habit strength. Overall, participants reported that WatchGuardian had better usability (SUS: 73.3 ± 12.8) than the baseline (66.8 ± 15.9), with significance through a Wilcoxon rank sum test (p < 0.05). WatchGuardian achieved a SUS score over 70, indicating acceptable usability. In both methods, false positive notifications were inevitable and could introduce participants’ confusion or surprise, which could explain the subpar SUS scores in general.

Interestingly, the results of WAI and habit strength did not indicate such a difference. Participants had similar reports of the relationship with the system (WAI score: 42.1 ± 7.1 for the WatchGuardian vs. 41.9 ± 9.3 for the baseline, p = 0.58). The change of habit strength between the pre- and post-intervention stages is also minimal (Δ of habit strength score: −4.2 ± 5.5 for the WatchGuardian vs. −5.5 ± 6.6 for the baseline, p = 0.25). This was probably due to the fact that the intervention sessions were not long enough to form a long-term alliance between users and the system, or to influence longitudinal behaviors or habits. Our qualitative results from semi-structured interviews provide more nuanced insights into these results.

### 5.6 Qualitative Results

All interviews were recorded and transcribed. We adopted a simple content analysis framework [111]. One author took extensive notes during the interviews, went through the scripts to categorize themes and count their frequency, and discussed with two other authors until convergence. We summarize our key findings below.

#### 5.6.1 Perception of AI-Powered Intervention.

Multiple participants reported that the AI-powered system possessed a sense of presence or “*having a soul*.” For instance, P10 noted, “*[WatchGuardian] resembles a habit instructor, or even like my mom… who would gently remind me when I scratch my head*.” P18 remarked, “*This system seems to read my mind, anticipating when I’m about to bite my lips and reminding me just in time. Sometimes I felt like I was sneaking around when making these actions*.” Compared to the rule-based condition, WatchGuardian’s interventions appeared to foster greater self-reflection among users. Notably, P19 even perceived the AI’s reminders as rewards: “*After being caught [touching my face] several times initially, I managed to control myself for a while. Then, even if the system reminded me again, I felt it was affirming my progress, like receiving a reward*.” In contrast, the rule-based condition yielded opposite effects, “*This mode of notification felt random to me—it was just like a machine*” (P02).

However, some participants also had a negative experience with WatchGuardian, especially when it did not detect the actions accurately (mostly false positive). For example, P08 mentioned that WatchGuardian had limited impact, and that they also felt a sense of distrust. “*At first, when it reported errors a few times, I tried to look for reasons elsewhere. But it kept making mistakes, which became frustrating. When it occasionally got something right, I thought it was just luck!*” Participants could lose trust in WatchGuardian when the system made mistakes at the beginning of their interaction. This is supported by prior research in other human–AI interaction systems [28, 53, 142].

#### 5.6.2 Illusory Amplification of Intervention Strength.

We noticed a surprisingly interesting phenomenon: Several users (P09, P10, and P16) reported that the vibration strength of the AI-based intervention in WatchGuardian felt stronger than that of the rule-based intervention. However, the vibration setup was identical in the two sessions. Even after we explained the specific intervention methods after the two study sessions, P16 stated, “*Not only did I subjectively feel that Mode B [our WatchGuardian method] gave me a stronger sense of motion restraint, but it also seemed to vibrate more intensely. Are you sure it’s really the same sening?*” This indicated that participants might develop an illusory or distorted perception of the intervention’s strength when the interventions were delivered just-in-time. This feeling resembles the self-awareness enhancement reported in studies of the Watching Eyes Effect [22]. WatchGuardian may have functioned as a third-party observer, inducing a sense of being watched. This, in turn, could have increased users’ sensitivity to bodily sensations and their self-focused attention on self-presentation. We discuss this more in Section 6.1.

#### 5.6.3 Diverse Patterns of Human–AI Collaborative Relationship.

Users exhibited diverse patterns of engagement with the AI system. Some participants demonstrated adaptive behavioral modification in response to WatchGuardian’s reminders. As P14 described, “*Every time I shook my leg, it would remind me, which made me increasingly hesitant to move*.” This was aligned with our original design goal of introducing AI-powered JITI.

Other than reducing the target actions, we also observed other behavior patterns. One pattern emerged where participants developed an interesting competitive relationship with the AI for user agency. For instance, P8 articulated this sentiment: “*I wanted to compete with it—I tried to resist the urge just so it wouldn’t catch me*.” This competitive spirit evolved into experimental behavior for some users, who attempted to understand and control the system’s underlying logic. P18’s experience exemplified this progression: “*Initially, I felt caught red-handed with every reminder. Later, I noticed it wouldn’t always detect my subtle movements, so I started experimenting with the notification logic, trying to gain control over the reminders. Eventually, though, I made peace with it and lost the urge to perform the action altogether*.” These participants wanted to gain better agency in this human–AI relationship.

In addition, some participants developed playful interactions with the system, treating it as an engaging companion rather than a mere monitoring tool. For example, P17 shared: “*When the video was boring… I just wanted to goof around a bit. This thing was actually keeping an eye on me, so I’d mess with it for fun, play around with it, and boom—it would react right away. Kinda helped wake me up a bit? It was basically like playing a game*.” Overall, these diverse patterns between users and WatchGuardian suggest a set of potential collaborative relationships between the two sides. We discuss this finding in Section 6.2.

#### 5.6.4 Tolerance for Model Mistakes.

Despite potentially slightly lower accuracy in the real-world experiment compared to offline evaluation, many users did not perceive this as leading to a significant negative experience. P10 stated, “*Because false positives were infrequent… and the 90-minute experiment was actually quite long, any impression of them might have faded*.” P21 also found the impact to be minimal, explaining, “*You can just dismiss this with one tap. I frequently make accidental touches on my phone, so I’m accustomed to such things, and it doesn’t really bother me*.”

Some users even perceived that false positives from our WatchGuardian mode could serve as a positive reminder, an effect not observed with the rule-based mode. “*It’s actually more like an alarm clock*.” As P14 mentioned, “*Even if it doesn’t ’notice’ you at the exact right time, you might still feel like it just gave you a reminder*.” P11 also mentioned, “*Previously, with mode B [(our WatchGuardian method)], even when it reminded me after I had stopped the action, I felt like it was constantly watching me. However, this time with the mode A [rule-based mode], because the reminders weren’t timely or accurate, neither its false positives nor the system as a whole felt as effective to me*.”

Thus, although WatchGuardian can exhibit more false positives and false negatives during real-life use, user feedback reveals a limited negative impact on the intervention experience.

#### 5.6.5 Experience in Longitude Study.

In addition, our proof-of-concept longitudinal study with three participants (referred as L1–L3) allowed us to gather valuable insights into how users experience and feel about WatchGuardian over extended periods. Overall, WatchGuardian remained effective throughout the longitudinal study. As participant L2 noted, “*It is just like a hardworking and tireless monitor*.” Meanwhile, in a more dynamic daily scenario involving a variety of behaviors, false alarms do occur. As L3 pointed out, “*When I transition from a period of stillness to a new action, it tends to trigger a false positive. Even regular activities, like typing, can sometimes cause this*.”

The longitude study led to the discovery of some new insights. We observed that any initial discomfort from feeling monitored gradually faded with continued use. For instance, L1 mentioned, “*When I first put on the watch, I felt a bit on edge and unfamiliar with it, knowing something was active and tracking me. But now, I felt more comfortable. I would say, Okay, let’s see what you’ve [the system] got for me this time*.” This suggests that as users wear the device longer, their familiarity with the system will potentially grow, and their relationship with it evolves to feel more like a partnership. As a result, the discomfort or anxiety stemming from being monitored is diminished.

The growing familiarity with the smartwatch also mitigated the negative impact of false positives, while correct alerts for personal behaviors consistently provided a pleasant influence. “*I think the most delightful surprise from this watch is its ability to detect unconscious behaviors*,” L1 remarked. “*After wearing it for a while, I could generally anticipate when certain actions might trigger a false alarm, so the negative impact was reduced. But when I was shaking my leg, I often don’t notice it. The watch’s alert at that moment would be a genuine surprise, and that sense of pleasant surprise always remained during these 3 days*.” L2 shared a similar sentiment: “*Regarding these mistaken reminders, at first, it felt weird. Later, though, I came to see them as gentle prompts… they just reinforce my motivation and persistence to maintain my good record of avoiding that behavior*.”

Meanwhile, our long-term study also identified drawbacks in the current design, primarily concerning the system’s potential distraction and long-term evolution. L1 shared, “*While the current design works for casual activities like watching videos or phone browsing, it’s disruptive when I’m trying to focus on work, reading, or productivity, hindering my ability to get into a flow state*.” L2 also suggested improvements of identifying the progress of users: “*I feel there are some improvements needed before I can use this app for real life, especially enabling the system to learn better about what I want over time… rather than just repeating the same alert patterns*.” We discuss more future improvement directions in Section 6.

## 6 Discussion

In this work, we propose to leverage few-shot learning to enable users to self-define personal undesirable actions for personalized intervention on smartwatches. We developed a three-stage pipeline that began with a self-supervised, pre-trained IMU model for robust feature extraction, then fine-tuned it for accurate HAR, and finally enhanced it with data augmentation and synthesis that enabled rapid customization of new user-defined actions using only a small number of examples. We implemented this pipeline on a smartwatch as a real-time intervention system, WatchGuardian, and demonstrated its effectiveness and advantages over the rule-based method through a multi-hour user study. In this section, we discuss some interesting takeaways from our study, together with our vision of how WatchGuardian can be generally applied to other health domains. We also briefly summarize the limitations of our work.

### 6.1 Distorted Perception with AI-Powered Intervention

During the study, we observed an interesting phenomenon where some participants developed a distorted perception towards their own actions or the intervention (see Section 5.6). For instance, several participants felt WatchGuardian’s vibrations were stronger than the baseline (yet the actual strength of vibration remained constant), and some felt they did the target actions more frequently with WatchGuardian (yet the objective data indicated otherwise). There are several potential interpretations of such interesting observations. The distorted perception might be caused by participants’ heightened awareness of the AI-guided interventions: because WatchGuardian more accurately and promptly caught the target actions, users started to pay extra and prolonged attention to any intervention. This could leave a stronger impression on them, and subsequently, they found it stronger or more frequent. Another plausible explanation is that WatchGuardian’s detection accuracy and just-in-time reminders resemble those from family members or friends, imbuing the system with a sense of aliveness. Such human-like monitoring may also lead users to feel as though the system were ‘observing’ them, inducing an effect reminiscent of the Observer Effect, which increases self-focus and enhances exteroceptive somatosensory perception [7, 29, 109]. Meanwhile, the feeling of being observed tends to increase participants’ prosocial choices, which might also be one of the reasons for the reduction of undesired actions [12]. Another potential explanation is that the participants, often associating their personal and idiosyncratic undesirable actions with “wrong-doing” and thus responding with negative emotions, might have subconsciously perceived their undesirable actions as being more frequent due to the WatchGuardian’s more precise and timely feedback eliciting stronger negative emotions. This, combined with an emotional interpretation of being ’corrected’, may have amplified their perception of the intervention’s intensity (vibration strength) and created the mistaken impression of performing these actions excessively.

The differing performance across engagement levels suggests that WatchGuardian’s high accuracy enables compliance even during high-focus states, whereas rule-based interventions exhibit fluctuations that align with established findings on attention and receptivity. Specifically, prior work indicates that activity type constrains intervention availability [127] and that tasks demanding higher attentional engagement can impair the processing of irrelevant notifications [20], whereas low-engagement tasks allow for greater interruptibility [108]. Conversely, the sustained effectiveness of WatchGuardian implies that AI-powered reminders can intervene successfully despite limited cognitive resources while being perceived as meaningful—an interpretation echoing the feedback in Section 5.6.1, where participants contrasted rule-based interventions as mere “machines” with WatchGuardian’s ability to “read minds.” This also suggests that adjustments to the reminder strategy call for a more context-aware design, aiming to achieve positive health benefits without causing information overload or diminishing productivity [85, 90]. Meanwhile, it is an interesting open question of how long such perception will last from a longitudinal intervention perspective. Depending on the cases, the growing self-awareness and/or reliability of AI could potentially assist users in building a long-term habit to reduce the target action, or on the contrary, the effect may fade away due to the AI intervention method no longer being novel or enticing. Future work can explore the lasting effect of the intervention, the corresponding perception, as well as user engagement in a long-term, field-based intervention study. [93, 131, 153].

### 6.2 Towards Human–AI Collaborative Interventions

Users’ mental models of WatchGuardian varied significantly. Some viewed it as a passive watchdog, and some viewed it as a playful interactive system, while others sought to take greater agency in the moment of intervention delivery. Our findings show the potential for and benefit of developing a collaborative relationship between humans and AI for behavioral intervention—“human–AI collaboration [150].” An AI system can provide appropriate support to users and help them achieve effective intervention outcomes. Such collaboration is closely relevant to the vision of just-in-time adaptive interventions [98, 99], where the delivery timing and methods of intervention are designed to be dynamically adapting to an individual’s internal state and surrounding context.

For instance, for users who see the system as a passive monitor, the system can provide the option for them to configure the frequency and style of intervention (e.g., higher/lower-intensity vibrations or consolidated notifications), ensuring the AI remains in the background but still provides supportive nudges. Taking one step further, the AI system may analyze user behavior over time and suggest new setups or goals for users with transparency (e.g., transitioning from nail-biting to managing stress). Users can accept, modify, or reject these suggestions, creating a dialogue where AI acts as a coach or collaborator rather than a rigid enforcer of pre-defined behaviors. Meanwhile, for those who see AI as a proactive system, one promising avenue is to incorporate user feedback into the AI’s learning process [104]. Users can label the AI’s predictions as accurate or not, which could serve as input for the model to further adapt to the user and improve performance over time (e.g., through reinforcement learning). Combined with contextual information that can potentially be inferred from sensors [162], such feedback can enable more precise, context-sensitive and personalized JITIs. In addition, the system would periodically prompt users to reassess their goals and update intervention targets, ensuring long-term relevance and efficacy.

It is noteworthy that such a human–AI collaboration paradigm needs to follow the principles of transparency and ethical design. Other than the options mentioned above, namely custom configurations and continuous feedback, users should have visibility into the system’s functionality and action logic regardless of the specific collaboration setup. This is important to provide users with agency and build their trust in the system.

### 6.3 Beyond Smartwatch and Broader Customization

In this work, our real-time intervention was implemented on a smartwatch. However, our proposed idea of empowering users to define any personal action and achieve a personalized intervention system can be more broadly applied to other domains. Instead of relying solely on a watch-based IMU, we can explore other body-based sensor arrays (e.g., headbands, rings, or joint sensors) to capture a more diverse range of behaviors in real time. This would enable the system to accommodate a wide variety of undesirable actions or habits, such as posture corrections and fidgeting management. In addition, beyond physical interventions, future customization can also delve into psychological or mental health support. For instance, individuals dealing with obsessive-compulsive disorder or other habitual thought/action patterns could define personal triggers (e.g., a particular repetitive motion or behavioral cue) and receive timely AI-driven interventions. Such holistic approaches highlight the flexibility and scalability of our pipeline. By enabling user-defined actions, we open up possibilities for long-term and effective management of both physical and psychological well-being using a multitude of wearable and sensor-based platforms.

### 6.4 Towards The Combination of Self-Tracking and JIT Intervention

Our approach is closely related to the field of personal informatics, which empowers users to monitor, record, and reflect on their own behaviors [31, 56, 74, 119]. Traditional self-tracking systems primarily focus on goal setting, retrospective reflection, and long-term habit awareness [56, 74], whereas JITI systems provide proactive, real-time guidance to influence user behavior [20, 99]. In this work, WatchGuardian enables users to define their personalized behaviors and receive real-time interventions based on immediate detection. This approach emphasizes customization and responsiveness rather than retrospective reflection based on long-term tracking of personal data. Future work could explore integrating long-term self-tracking data with real-time interventions. For example, systems could aggregate historical behavior data to provide visualizations of action frequency, trends, and progress over time [152], or to adjust intervention strategies based on long-term patterns [104]. Such integration would combine immediate intervention with longitudinal insights, supporting reflection and more effective habit formation.

### 6.5 Limitations and Future Work

Despite WatchGuardian’s positive outcome and the promising insights generated, we recognize some limitations.

#### 6.5.1 Technical Modeling and Implementation.

Above all, current model relies solely on accelerometer data for action recognition, which may limit its ability to capture the full range of motion characteristics or other physiology. Future work can explore additional sensing modalities, such as gyroscope, photoplethysmography, and joint locations, to enhance the accuracy and robustness of action recognition.

Besides, our study employed a remote server for model training and inference to access greater computational resources. Cloud-based processing remains the predominant paradigm in academic research [104, 161] and in commercial wearable systems [21, 34, 50, 86]. However, network communication between mobile devices and remote servers introduces overhead, as it increases latency, limits real-time responsiveness, and incurs additional risks. This design necessitates stable internet access for data transmission, which may hinder usability in low-connectivity environments. It also raises privacy concerns, as the continuous transmission of IMU data to an external server could expose sensitive personal information if the communication is intercepted or compromised. While none of the participants in our study explicitly expressed privacy concerns, prior work has shown that IMU signals can reveal behavioral and physiological patterns [67]. Therefore, although this setup was sufficient for our proof-of-concept study, future work should explore secure data transmission mechanisms [17, 21] and investigate on-device inference strategies, potentially leveraging lightweight models and hardware-accelerated frameworks such as TensorFlow Lite [80] or Core ML [49] to reduce computational cost and eliminate reliance on client-server communication [38, 124].

#### 6.5.2 Intervention Evaluation.

Our study involved 21 participants in the short-term user study and 3 in the longitudinal phase. Given this sample size, our findings may not fully capture the variability and diversity of human activities in real-world scenarios [101, 144]. Future work engaging a broader population could therefore offer more diverse insights and validate these preliminary results. Regarding the experimental setup, we required participants to wear the smartwatch on their dominant wrist, as the dominant hand typically exhibits more complex motor patterns [66]. Furthermore, its superior dexterity facilitates interaction and enables better capture of the fine-grained undesired actions targeted in this work [26]. Acknowledging that smartwatches are more commonly worn on the non-dominant arm, we believe that investigating our framework in such settings is a valuable avenue for future research.

In terms of the evaluation scope, the current evaluation focused on adding one customized action per user. Although our offline performance evaluation of the few-shot pipeline demonstrated the potential to support multiple customized actions, the scalability of the framework to handle multiple or simultaneous actions (e.g., nail-biting and leg-shaking) remains to be validated in future work. Additionally, although we tried to simulate real-life scenarios, our intervention study was conducted over a limited duration and in controlled experimental settings, which may not fully reflect the complexities and dynamics of real-life environments. Different postures (such as standing or walking, beyond sitting) may also affect sensor readings and classification stability, which were not systematically tested in this study. We conducted a small-scale, proof-of-concept study with three participants to collect qualitative feedback, but it only revealed limited insights. Real-world contexts introduce factors such as environmental noise, varying sensor placements, and user behavior changes over time [91, 94, 144, 145], which were not thoroughly studied in this study. Future research should conduct longitudinal field experiments with real-world deployment of the system.

## 7 Conclusion

In this study, we introduced WatchGuardian, a smartwatch-based intervention system that empowers users to define and reduce their own personal and idiosyncratic undesirable actions. WatchGuardian employs a three-stage, few-shot learning pipeline to recognize newly defined actions with only a small number of samples. Through an extensive evaluation of the model’s offline performance and an intervention study, our findings demonstrate that WatchGuardian achieves robust and data-efficient action recognition but also significantly decreases users’ undesirable actions compared to a rule-based baseline. Our findings underscore the potential of personalized, AI-driven JITIs for individuals seeking to mitigate personal habits and behaviors. We envision that our work offers a versatile foundation for creating broader, user-defined intervention systems that leverage advanced AI solutions to accommodate an ever-growing spectrum of personal needs.

## Supplementary Material

Supplementary Material

## Figures and Tables

**Fig. 1.**
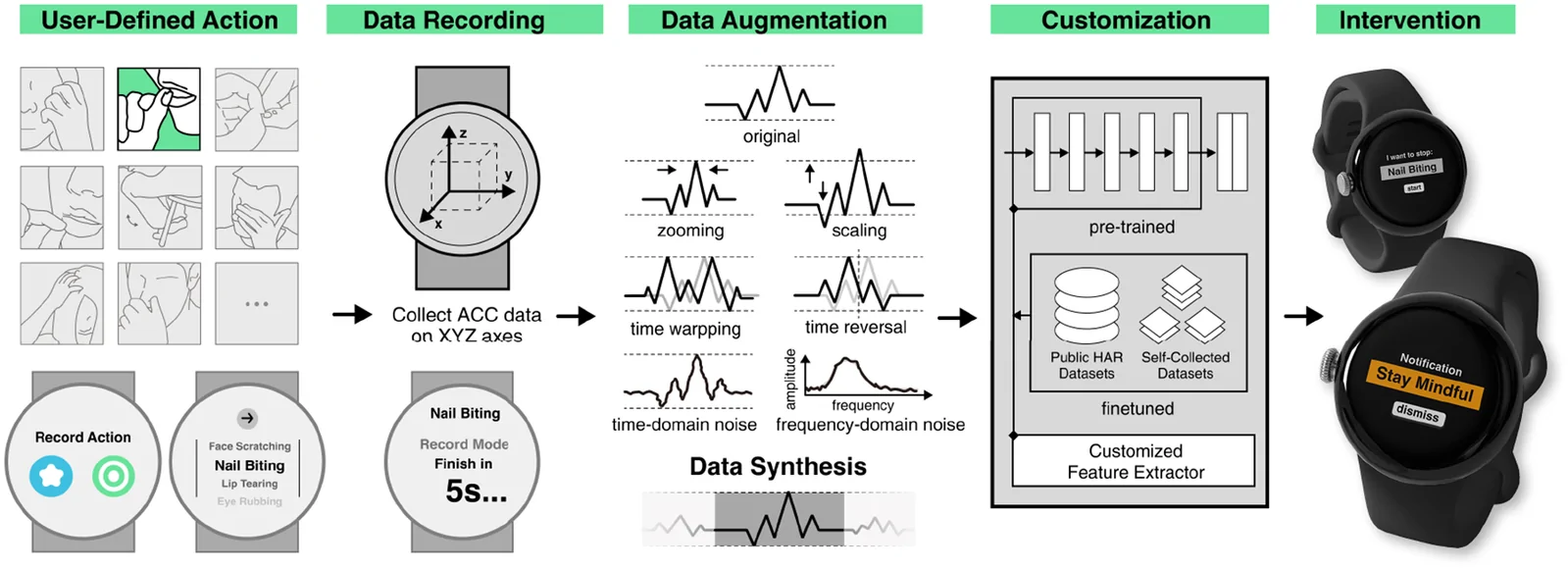
WatchGuardian empowers users to easily define personal actions that they want to receive JITI from a smartwatch. The user journey is as follows: (1) Users determine one or more custom target actions. (2) They follow the instructions on the smartwatch to collect a small set of samples with the accelerometer sensor. (3) WatchGuardian applies multiple data augmentation and data synthesis techniques to expand the training dataset. (4) WatchGuardian adapts a pre-trained model through fine-tuning and personal customization. (5) WatchGuardian leverages the custom model to provide a JITI system for real-time action recognition and intervention delivery. JITI, Just-in-time intervention.

**Fig. 2.**
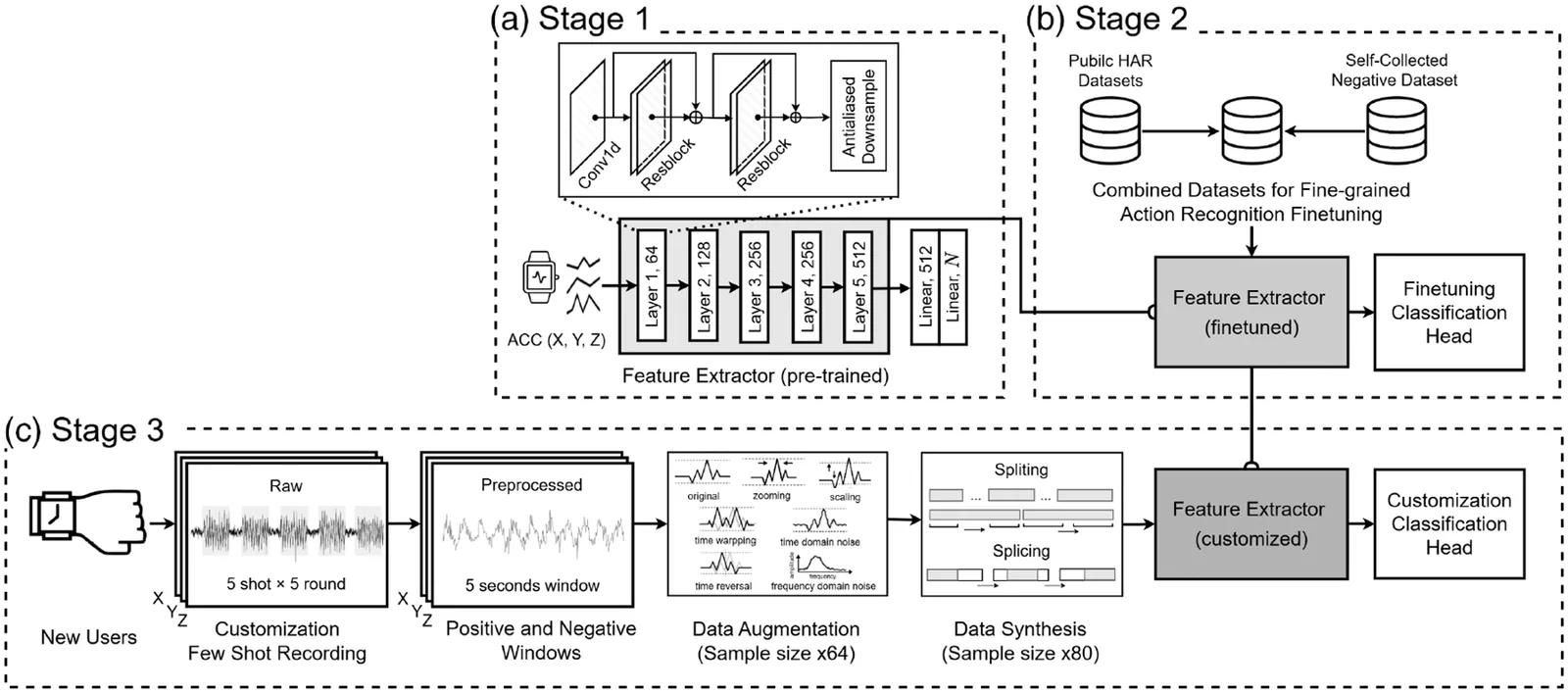
Three-stage few-shot pipeline for model customization. (a) Stage 1: We adopted a pre-trained SSL model for HAR that takes 30 Hz tri-axis accelerometer data streams. (b) Stage 2: We fine-tuned the pre-trained model on two HAR datasets with more fine-grained gestures, together with additional negative data collected by us. (c) Stage 3: Given the data sequence of a few samples of the new target action, we designed a series of data augmentation and synthesis techniques to enable robust modeling training for customization.

**Fig. 3.**
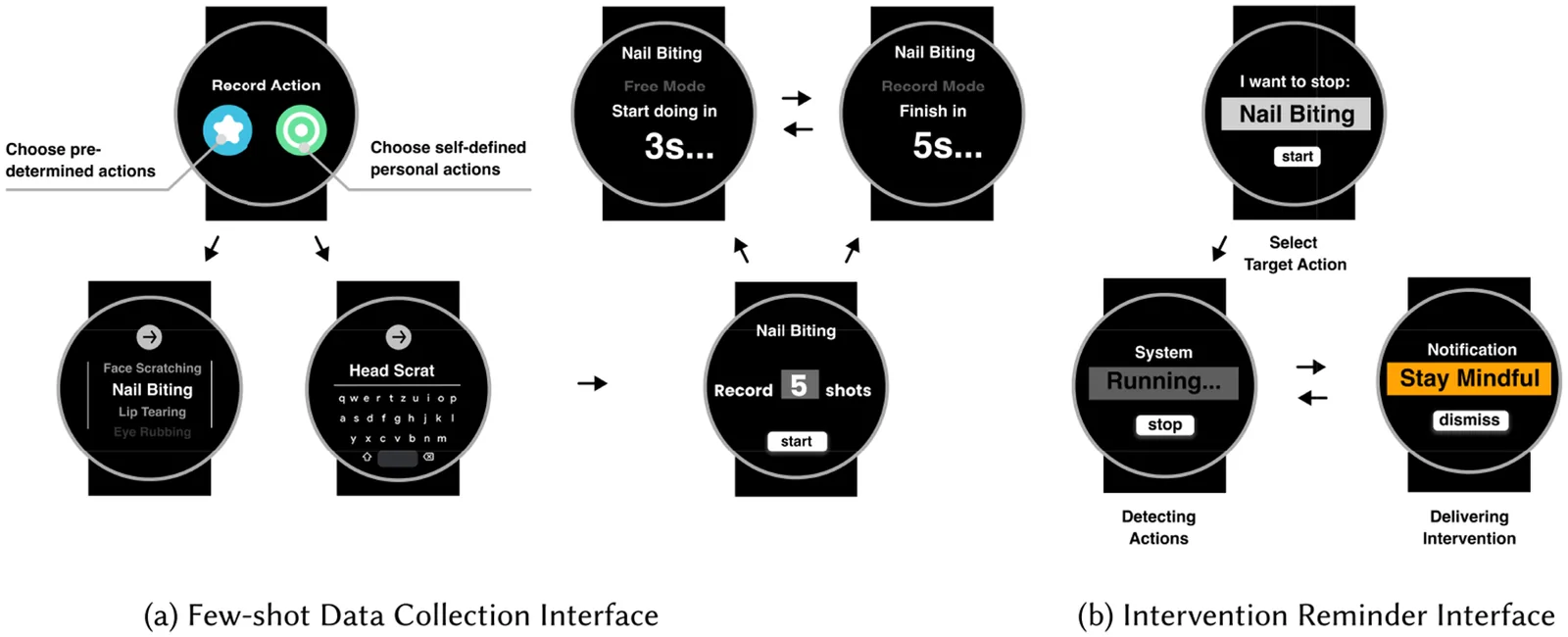
Smartwatch interface designs. (a) Few-shot data collection interface, where a user can define the target behavior and the number of shots. The user can name the gesture once the collection is finished. (b) Intervention reminder interface, which is shown when the system detects undesirable target actions.

**Fig. 4.**
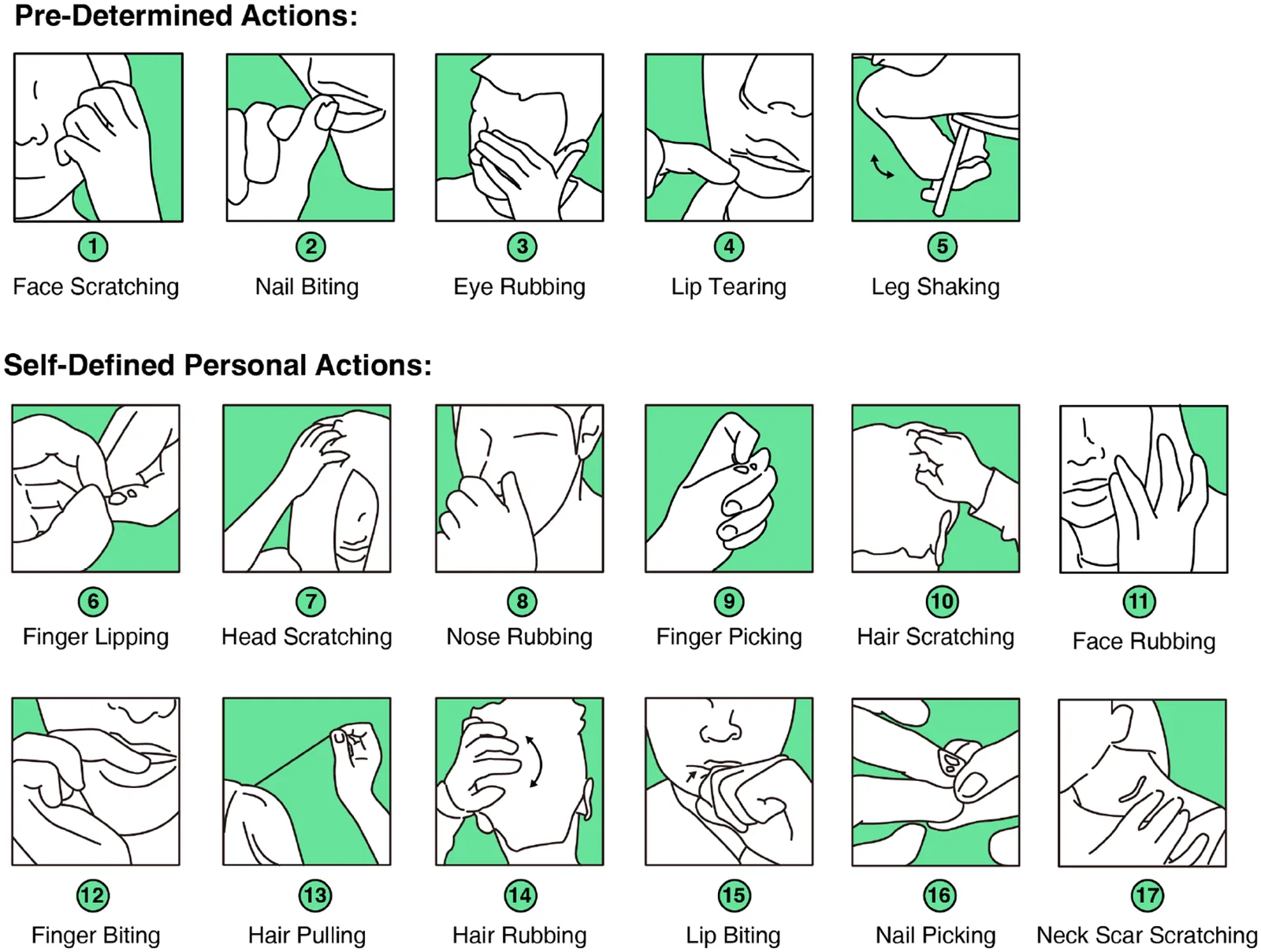
Target actions for evaluation. Subfigures (1–5) present the five pre-determined actions. Subfigures (6–17) visualize new target behaviors defined by participants. Only identical actions are grouped as one. Actions that have minor differences are counted separately, as each of them could be highly personal.

**Fig. 5.**
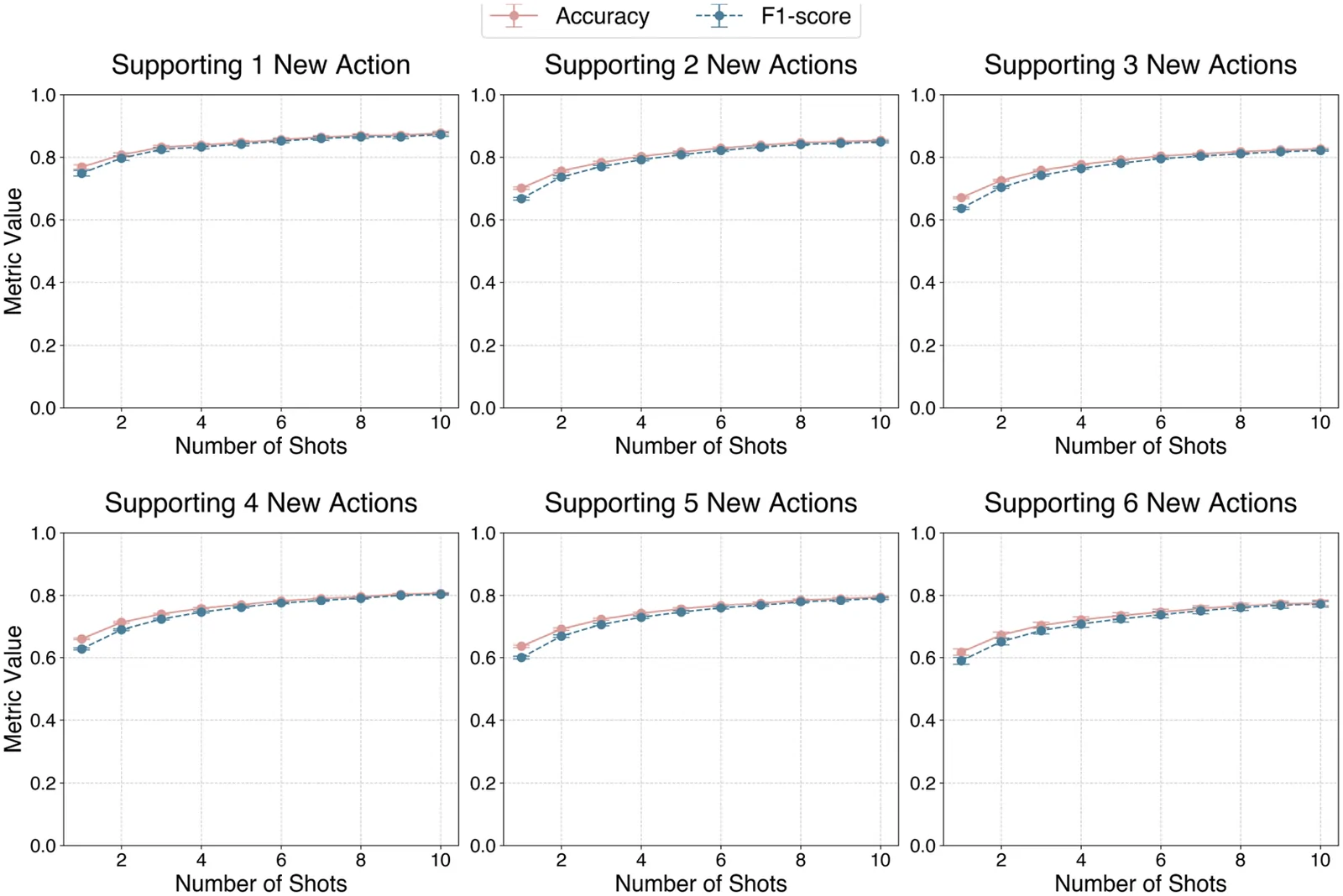
Few-shot learning pipeline performance of accuracy and F1 score. We experimented with different numbers of shots using 1–10 samples to train a custom model. We also experimented with adding more than one target action simultaneously (i.e., multi-class classification). Error bars indicate standard error. The same below.

**Fig. 6.**
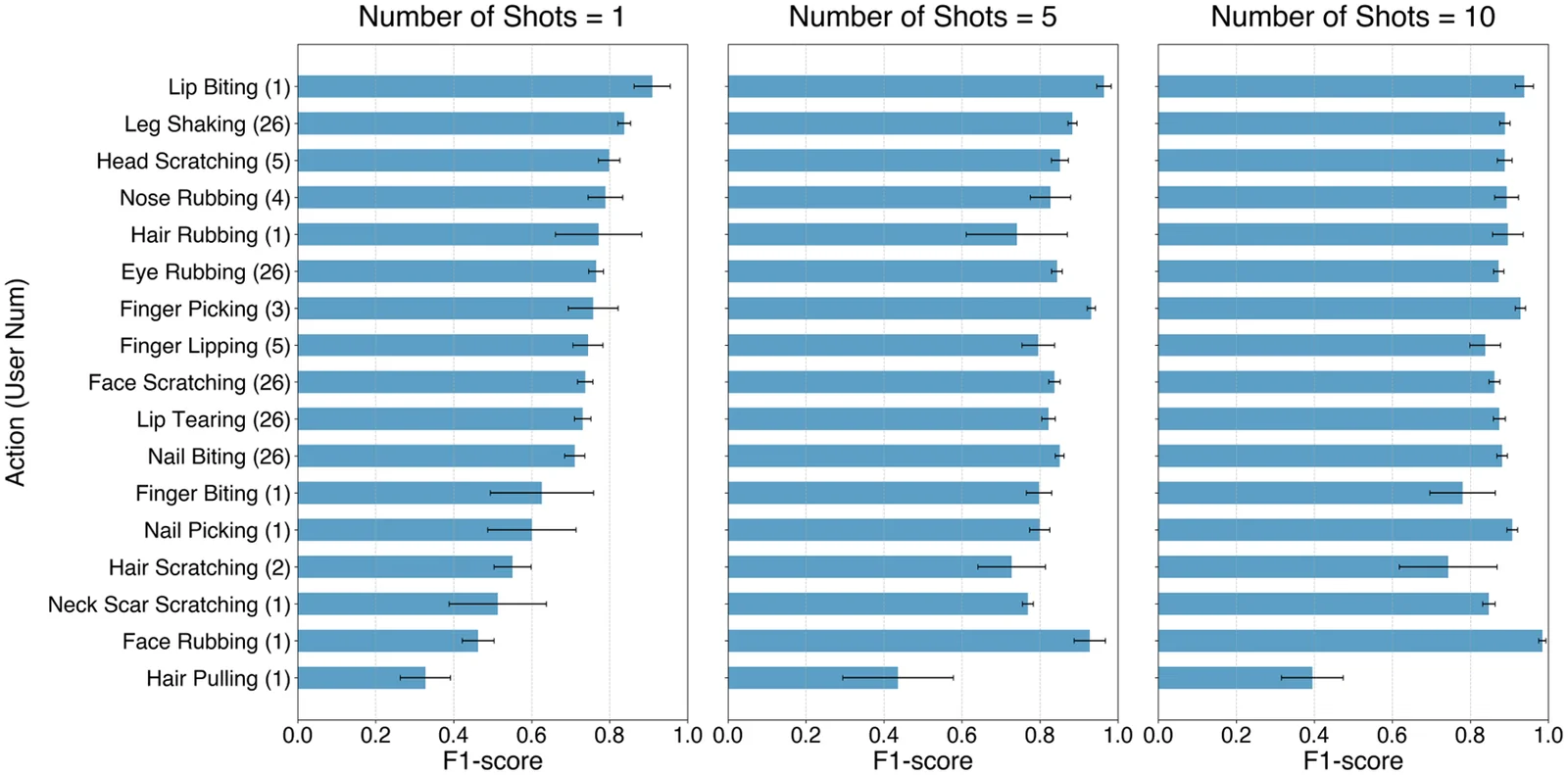
Model performance of recognizing each action with 1, 5, or 10 shots. For consistency, each action was added alone (i.e., binary classification model). The “(User Num)” indicates how many users did this action. The five pre-determined actions (Lip Tearing, Nail Biting, Face Scratching, Eye Rubbing, and Leg Shaking) have the total number of participants (26), and other self-defined actions are more scattered.

**Fig. 7.**
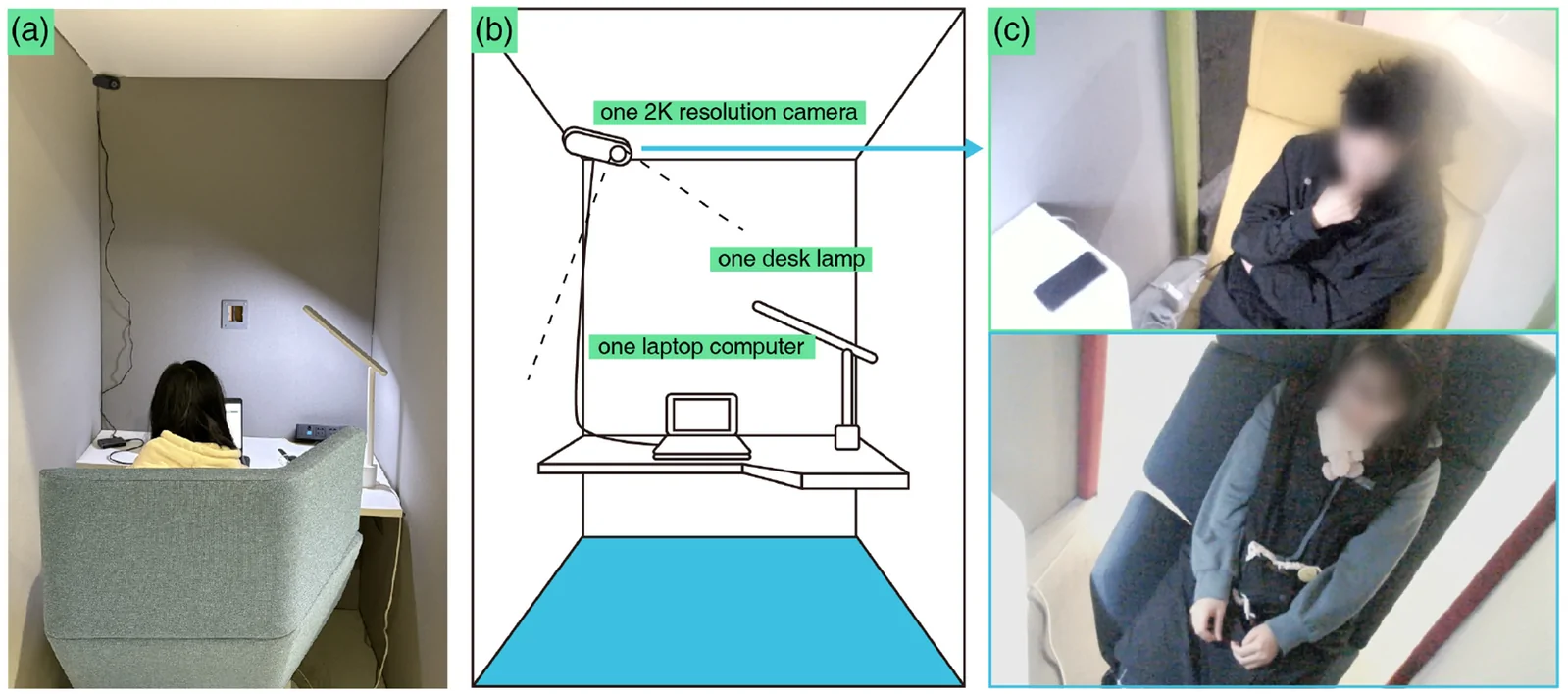
WatchGuardian intervention evaluation setup. (a) The photo of a participant in the room. (b) The sketch of the study room and apparatus setup for intervention. (c) The video from the camera on the corner that records the ground truth.

**Fig. 8.**
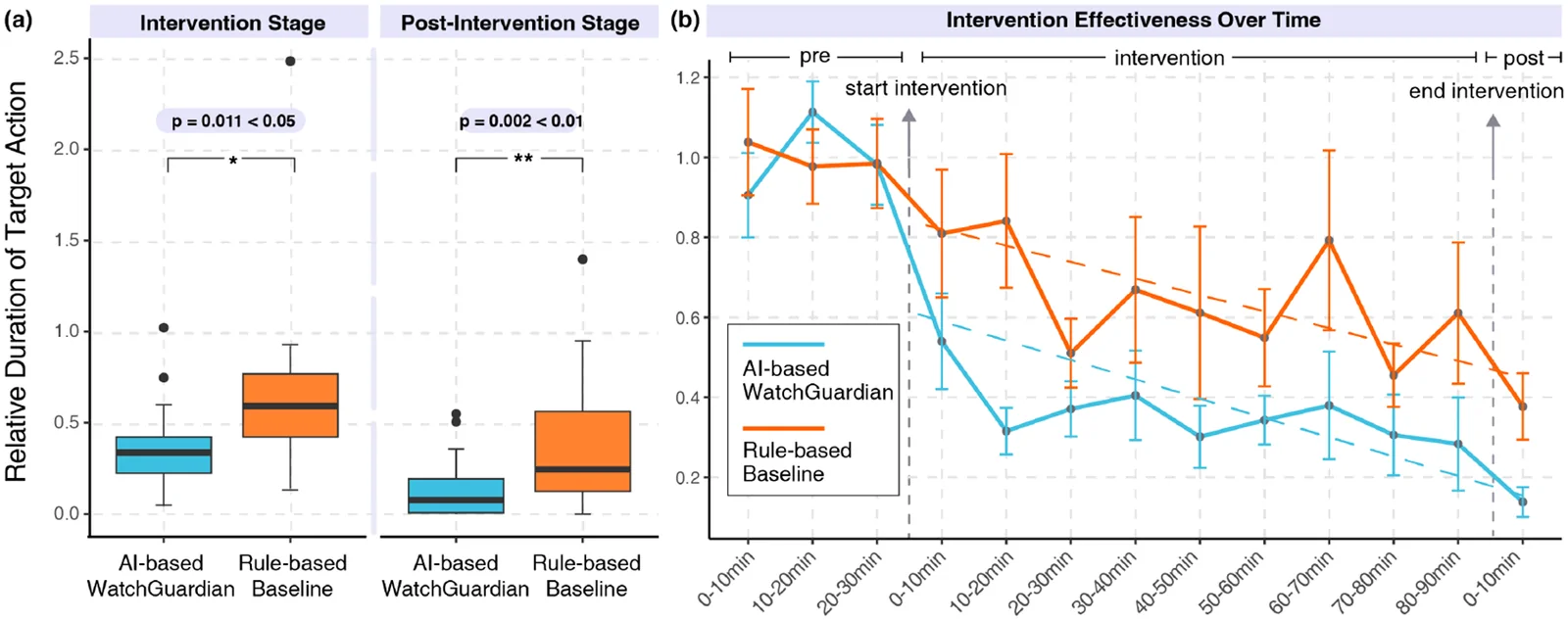
(a) Relative duration of target action every 10 minutes in intervention and post-intervention stages (compared to the pre-intervention stage). A number lower than 1.0 means that an individual performed fewer target actions after intervention. (b) Average relative duration of target action over time. The dashed lines fit the last 10 minutes of the pre-intervention stage and the rest of the session.

**Fig. 9.**
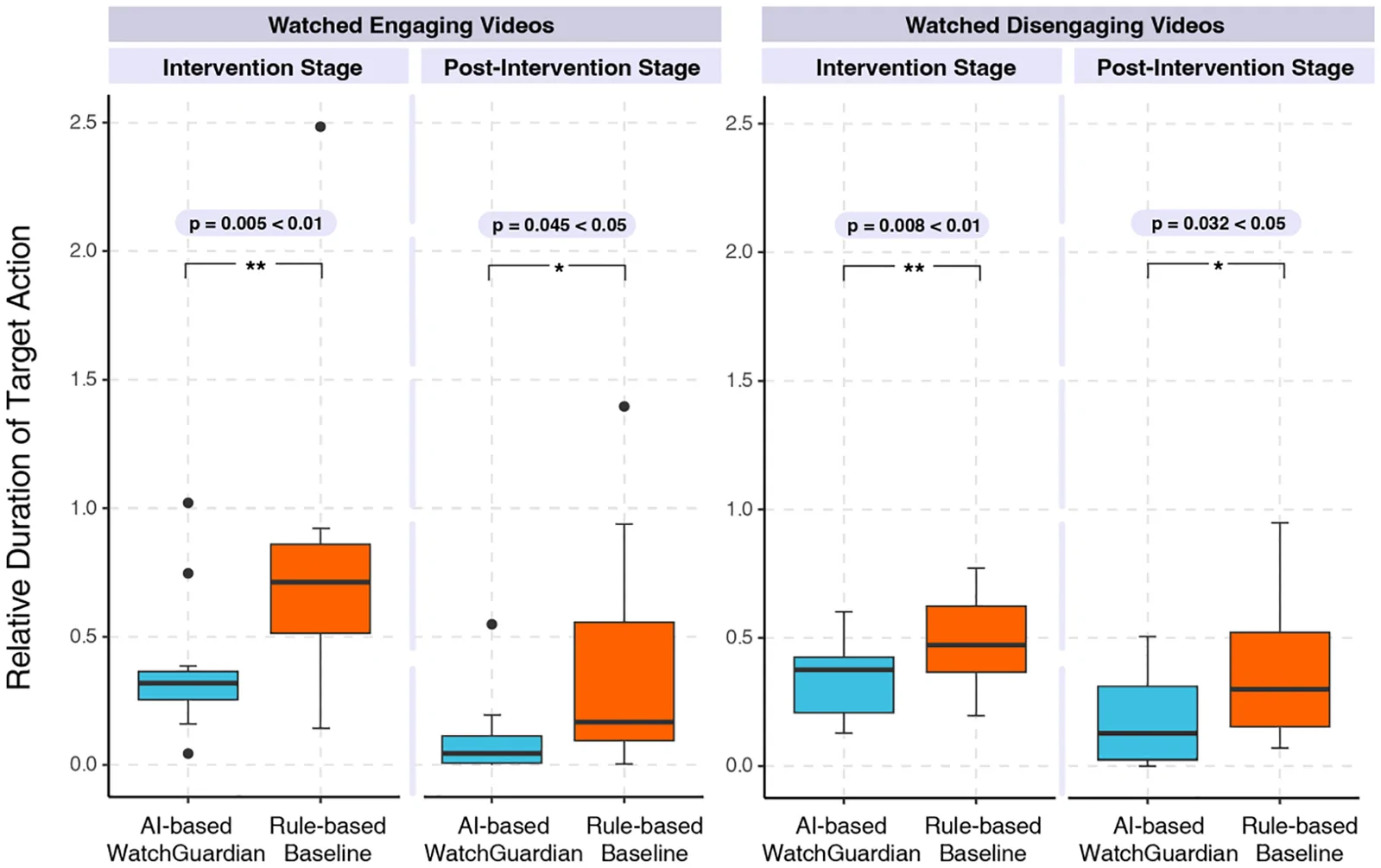
(a) Relative duration of target action for participants who watched engaging videos. (b) Relative duration of target action for participants who watched disengaging videos.

**Table 1.** Detailed Few-Shot Pipeline Performance with Different Numbers of Shots When Adding One Personal Action

Shots	Window-level				Action-level			
	Acc	Prec	Rec	F1	Acc	Prec	Rec	F1
1	0.614 ± 0.006	0.700 ± 0.007	0.614 ± 0.006	0.571 ± 0.008	0.768 ± 0.007	0.810 ± 0.007	0.768 ± 0.007	0.748 ± 0.009
3	0.658 ± 0.005	0.736 ± 0.005	0.658 ± 0.005	0.634 ± 0.006	0.832 ± 0.006	0.860 ± 0.005	0.832 ± 0.006	0.825 ± 0.006
5	0.670 ± 0.005	0.746 ± 0.005	0.670 ± 0.005	0.648 ± 0.006	0.847 ± 0.005	0.871 ± 0.005	0.847 ± 0.005	0.842 ± 0.006
7	0.685 ± 0.005	0.755 ± 0.005	0.685 ± 0.005	0.667 ± 0.006	0.864 ± 0.005	0.883 ± 0.005	0.864 ± 0.005	0.860 ± 0.006
10	0.702 ± 0.005	0.763 ± 0.005	0.702 ± 0.005	0.688 ± 0.006	0.877 ± 0.005	0.890 ± 0.005	0.877 ± 0.005	0.873 ± 0.006

Window-level results are based on each sliding window as a data point. Action-level results are the aggregation of the sliding windows after smoothing post-processing (threshold = 3) and are closer to real-life application scenarios.

**Table 2.** Action-Level Results of Comparison Study and Ablation Study

Methods	Acc	Prec	Rec	F1
**SVM**	0.790 ± 0.007	0.843 ± 0.006	0.790 ± 0.007	0.750 ± 0.009
**Random Forest**	0.796 ± 0.007	0.843 ± 0.007	0.796 ± 0.007	0.761 ± 0.010
**COVID-away** [135]	0.702 ± 0.004	0.779 ± 0.006	0.702 ± 0.004	0.644 ± 0.006
**Itchtector** [69]	0.802 ± 0.005	0.847 ± 0.004	0.802 ± 0.005	0.782 ± 0.006
**HandGesCus** [152]	0.704 ± 0.009	0.677 ± 0.014	0.704 ± 0.009	0.648 ± 0.013
**SVM (data augmentation and synthesis)**	0.729 ± 0.006	0.793 ± 0.006	0.729 ± 0.006	0.676 ± 0.008
**Random Forest (data augmentation and synthesis)**	0.721 ± 0.010	0.737 ± 0.013	0.721 ± 0.010	0.670 ± 0.012
**Itchtector (data augmentation and synthesis)**	0.726 ± 0.007	0.772 ± 0.008	0.726 ± 0.007	0.662 ± 0.009
**WatchGuardian w/o all**	0.704 ± 0.006	0.770 ± 0.006	0.704 ± 0.006	0.662 ± 0.008
**WatchGuardian with Stage 1 (pre-trained SSL model)** [158]	0.787 ± 0.006	0.833 ± 0.005	0.787 ± 0.006	0.766 ± 0.007
**WatchGuardian with Stage 1 and 2 (fine-tuning)**	0.789 ± 0.006	0.830 ± 0.005	0.789 ± 0.006	0.772 ± 0.008
**WatchGuardian with Stage 1, 2, and 3 (data augmentation and synthesis)**	**0.847 ± 0.005**	**0.871 ± 0.005**	**0.847 ± 0.005**	**0.842 ± 0.006**

The same training (five shots, one new target behavior) and testing data were used to ensure consistency.

## CCS Concepts:

• **Human-centered computing** → **Ubiquitous and mobile computing**; • **Applied computing** → **Life and medical sciences**;

## Footnotes

1 See the model and implementation at: https://github.com/OxWearables/ssl-wearables.

2 The two datasets are available at https://zenodo.org/records/7058383 and https://doi.org/10.18738/T8/NNDFQD.

3 The system operates in the background without impacting other smartwatch functions. However, since Android’s battery optimization strategy precludes stable background data transmission unless the screen remains on, our usage test on the Google Pixel Watch 2 yielded approximately 6 hours of battery life. The accelerometer has a maximum power of approximately 2 mW [112]. Therefore, we anticipate that, if data transmission is enabled while the screen is off, the watch will have substantially longer battery life.

4 In real-world applications, we envision that such negative data can also be passively collected and implicitly embedded in the instruction process, thereby introducing minimal additional workload for the user.

5 Several participants also mentioned the scenarios under pressure or stress. Considering the feasibility and ethics of a multi-hour intervention study, we did not provide this option.

6 Due to the restrictions of the number of devices and limited battery life, we regretfully could not do a larger-scale longitudinal study. We recognize this as a limitation of our study in the discussion.

7 It’s challenging to reliably obtain ground truth in the longitudinal study. Here we only report the results of the 4-hour study, as it has video recordings as the ground truth.
