# Supplementary material for: WatchGuardian: Enabling User-Defined Personalized Just-in-Time Intervention on Smartwatch
Source: ACM Trans Comput Healthc. Author manuscript; Available in PMC 2026 Nov 11. (PMC13750837; doi:10.1145/3788689)
Supplement: Supplementary file 1 [file NIHMS2219703-supplement-Supplementary_Material.pdf]

A Few-shot Pipeline Performance with Different Numbers of Actions and Shots

Table 1. Prediction Performance with Different Number of Actions and Shots. (Action Number 1–3)

Actions	Shots	Window-level				Action-level			
		Acc	Prec	Rec	F1	Acc	Prec	Rec	F1
1	1	0.614±0.006	0.700±0.007	0.614±0.006	0.571±0.008	0.768±0.007	0.810±0.007	0.768±0.007	0.748±0.009
	2	0.628±0.005	0.710±0.006	0.628±0.005	0.620±0.006	0.800±0.006	0.835±0.006	0.800±0.006	0.810±0.006
	3	0.658±0.005	0.736±0.005	0.658±0.005	0.634±0.006	0.832±0.006	0.860±0.005	0.832±0.006	0.825±0.006
	4	0.664±0.005	0.743±0.005	0.664±0.005	0.641±0.006	0.839±0.005	0.866±0.005	0.839±0.005	0.833±0.006
	5	0.670±0.005	0.746±0.005	0.670±0.005	0.648±0.006	0.847±0.005	0.871±0.005	0.847±0.005	0.842±0.006
	6	0.676±0.005	0.749±0.005	0.676±0.005	0.657±0.006	0.856±0.005	0.879±0.005	0.856±0.005	0.852±0.006
	7	0.685±0.005	0.755±0.005	0.685±0.005	0.667±0.006	0.864±0.005	0.883±0.005	0.864±0.005	0.860±0.006
	8	0.691±0.005	0.758±0.005	0.691±0.005	0.674±0.006	0.869±0.005	0.887±0.005	0.869±0.005	0.865±0.006
	9	0.696±0.005	0.760±0.005	0.696±0.005	0.680±0.006	0.870±0.005	0.885±0.005	0.870±0.005	0.865±0.006
	10	0.702±0.005	0.763±0.005	0.702±0.005	0.688±0.006	0.877±0.005	0.890±0.005	0.877±0.005	0.873±0.006
2	1	0.578±0.003	0.622±0.004	0.578±0.003	0.521±0.004	0.701±0.004	0.717±0.004	0.701±0.004	0.667±0.004
	2	0.589±0.003	0.635±0.004	0.589±0.003	0.545±0.004	0.745±0.004	0.760±0.004	0.745±0.004	0.730±0.004
	3	0.639±0.003	0.684±0.003	0.639±0.003	0.608±0.003	0.783±0.003	0.797±0.003	0.783±0.003	0.770±0.003
	4	0.653±0.003	0.696±0.003	0.653±0.003	0.625±0.003	0.803±0.003	0.815±0.003	0.803±0.003	0.792±0.003
	5	0.667±0.003	0.707±0.003	0.667±0.003	0.643±0.003	0.817±0.003	0.827±0.003	0.817±0.003	0.808±0.003
	6	0.679±0.003	0.717±0.003	0.679±0.003	0.659±0.003	0.829±0.003	0.838±0.003	0.829±0.003	0.821±0.003
	7	0.690±0.003	0.725±0.003	0.690±0.003	0.672±0.003	0.838±0.003	0.847±0.003	0.838±0.003	0.832±0.003
	8	0.699±0.003	0.731±0.003	0.699±0.003	0.682±0.003	0.847±0.003	0.854±0.003	0.847±0.003	0.841±0.003
	9	0.704±0.003	0.735±0.003	0.704±0.003	0.689±0.003	0.850±0.003	0.858±0.003	0.850±0.003	0.845±0.003
	10	0.708±0.003	0.738±0.003	0.708±0.003	0.695±0.003	0.854±0.003	0.862±0.003	0.854±0.003	0.849±0.003
3	1	0.566±0.002	0.572±0.003	0.566±0.002	0.511±0.003	0.670±0.003	0.667±0.004	0.670±0.003	0.636±0.003
	2	0.587±0.002	0.605±0.003	0.587±0.002	0.555±0.003	0.723±0.003	0.730±0.003	0.723±0.003	0.710±0.003
	3	0.630±0.002	0.654±0.003	0.630±0.002	0.596±0.003	0.758±0.002	0.766±0.003	0.758±0.002	0.742±0.003
	4	0.647±0.002	0.671±0.003	0.647±0.002	0.617±0.003	0.777±0.002	0.785±0.003	0.777±0.002	0.764±0.003
	5	0.660±0.002	0.683±0.002	0.660±0.002	0.635±0.003	0.791±0.002	0.799±0.003	0.791±0.002	0.781±0.003
	6	0.673±0.002	0.694±0.002	0.673±0.002	0.650±0.003	0.804±0.002	0.812±0.002	0.804±0.002	0.795±0.003
	7	0.682±0.002	0.701±0.002	0.682±0.002	0.662±0.003	0.811±0.002	0.819±0.002	0.811±0.002	0.803±0.002
	8	0.690±0.002	0.709±0.002	0.690±0.002	0.672±0.003	0.817±0.002	0.826±0.002	0.817±0.002	0.811±0.002
	9	0.697±0.002	0.714±0.002	0.697±0.002	0.680±0.003	0.823±0.002	0.831±0.002	0.823±0.002	0.818±0.002
	10	0.701±0.002	0.718±0.002	0.701±0.002	0.686±0.003	0.826±0.002	0.835±0.002	0.826±0.002	0.822±0.002

Table 2. Prediction Performance with Different Number of Actions and Shots. (Action Number 4–6)

Actions	Shots	Window-level				Action-level			
		Acc	Prec	Rec	F1	Acc	Prec	Rec	F1
4	1	$0.557_{\pm0.002}$	$0.553_{\pm0.003}$	$0.557_{\pm0.002}$	$0.501_{\pm0.003}$	$0.659_{\pm0.003}$	$0.656_{\pm0.003}$	$0.659_{\pm0.003}$	$0.627_{\pm0.003}$
	2	$0.589_{\pm0.002}$	$0.608_{\pm0.003}$	$0.589_{\pm0.002}$	$0.555_{\pm0.003}$	$0.723_{\pm0.003}$	$0.730_{\pm0.003}$	$0.723_{\pm0.003}$	$0.710_{\pm0.003}$
	3	$0.619_{\pm0.002}$	$0.635_{\pm0.003}$	$0.619_{\pm0.002}$	$0.583_{\pm0.003}$	$0.739_{\pm0.003}$	$0.745_{\pm0.003}$	$0.739_{\pm0.003}$	$0.723_{\pm0.003}$
	4	$0.635_{\pm0.002}$	$0.651_{\pm0.003}$	$0.635_{\pm0.002}$	$0.604_{\pm0.003}$	$0.757_{\pm0.003}$	$0.766_{\pm0.003}$	$0.757_{\pm0.003}$	$0.745_{\pm0.003}$
	5	$0.647_{\pm0.002}$	$0.663_{\pm0.003}$	$0.647_{\pm0.002}$	$0.621_{\pm0.003}$	$0.770_{\pm0.002}$	$0.778_{\pm0.003}$	$0.770_{\pm0.002}$	$0.761_{\pm0.003}$
	6	$0.658_{\pm0.002}$	$0.673_{\pm0.003}$	$0.658_{\pm0.002}$	$0.635_{\pm0.003}$	$0.782_{\pm0.002}$	$0.791_{\pm0.002}$	$0.782_{\pm0.002}$	$0.775_{\pm0.003}$
	7	$0.667_{\pm0.002}$	$0.681_{\pm0.003}$	$0.667_{\pm0.002}$	$0.646_{\pm0.003}$	$0.790_{\pm0.002}$	$0.799_{\pm0.002}$	$0.790_{\pm0.002}$	$0.783_{\pm0.002}$
	8	$0.676_{\pm0.002}$	$0.689_{\pm0.003}$	$0.676_{\pm0.002}$	$0.657_{\pm0.003}$	$0.796_{\pm0.002}$	$0.805_{\pm0.002}$	$0.796_{\pm0.002}$	$0.791_{\pm0.002}$
	9	$0.684_{\pm0.002}$	$0.697_{\pm0.003}$	$0.684_{\pm0.002}$	$0.667_{\pm0.003}$	$0.803_{\pm0.002}$	$0.814_{\pm0.002}$	$0.803_{\pm0.002}$	$0.800_{\pm0.002}$
	10	$0.689_{\pm0.002}$	$0.701_{\pm0.003}$	$0.689_{\pm0.002}$	$0.673_{\pm0.003}$	$0.807_{\pm0.002}$	$0.817_{\pm0.002}$	$0.807_{\pm0.002}$	$0.803_{\pm0.002}$
5	1	$0.542_{\pm0.003}$	$0.524_{\pm0.004}$	$0.542_{\pm0.003}$	$0.481_{\pm0.004}$	$0.636_{\pm0.004}$	$0.618_{\pm0.005}$	$0.636_{\pm0.004}$	$0.600_{\pm0.005}$
	2	$0.584_{\pm0.003}$	$0.581_{\pm0.004}$	$0.584_{\pm0.003}$	$0.538_{\pm0.004}$	$0.691_{\pm0.004}$	$0.689_{\pm0.004}$	$0.691_{\pm0.004}$	$0.668_{\pm0.004}$
	3	$0.610_{\pm0.003}$	$0.612_{\pm0.004}$	$0.610_{\pm0.003}$	$0.573_{\pm0.004}$	$0.722_{\pm0.004}$	$0.724_{\pm0.004}$	$0.722_{\pm0.004}$	$0.705_{\pm0.004}$
	4	$0.629_{\pm0.004}$	$0.635_{\pm0.004}$	$0.629_{\pm0.004}$	$0.597_{\pm0.004}$	$0.741_{\pm0.004}$	$0.746_{\pm0.004}$	$0.741_{\pm0.004}$	$0.728_{\pm0.004}$
	5	$0.643_{\pm0.004}$	$0.649_{\pm0.004}$	$0.643_{\pm0.004}$	$0.614_{\pm0.004}$	$0.755_{\pm0.004}$	$0.762_{\pm0.004}$	$0.755_{\pm0.004}$	$0.745_{\pm0.004}$
	6	$0.654_{\pm0.004}$	$0.661_{\pm0.004}$	$0.654_{\pm0.004}$	$0.629_{\pm0.004}$	$0.767_{\pm0.004}$	$0.774_{\pm0.004}$	$0.767_{\pm0.004}$	$0.759_{\pm0.004}$
	7	$0.663_{\pm0.004}$	$0.670_{\pm0.004}$	$0.663_{\pm0.004}$	$0.640_{\pm0.004}$	$0.774_{\pm0.004}$	$0.782_{\pm0.004}$	$0.774_{\pm0.004}$	$0.768_{\pm0.004}$
	8	$0.672_{\pm0.004}$	$0.679_{\pm0.004}$	$0.672_{\pm0.004}$	$0.651_{\pm0.004}$	$0.785_{\pm0.004}$	$0.793_{\pm0.004}$	$0.785_{\pm0.004}$	$0.779_{\pm0.004}$
	9	$0.678_{\pm0.004}$	$0.685_{\pm0.004}$	$0.678_{\pm0.004}$	$0.659_{\pm0.004}$	$0.789_{\pm0.004}$	$0.798_{\pm0.004}$	$0.789_{\pm0.004}$	$0.784_{\pm0.004}$
	10	$0.684_{\pm0.004}$	$0.690_{\pm0.004}$	$0.684_{\pm0.004}$	$0.666_{\pm0.004}$	$0.795_{\pm0.004}$	$0.803_{\pm0.004}$	$0.795_{\pm0.004}$	$0.790_{\pm0.004}$
6	1	$0.533_{\pm0.009}$	$0.510_{\pm0.010}$	$0.533_{\pm0.009}$	$0.479_{\pm0.010}$	$0.617_{\pm0.010}$	$0.613_{\pm0.011}$	$0.617_{\pm0.010}$	$0.589_{\pm0.011}$
	2	$0.579_{\pm0.008}$	$0.565_{\pm0.010}$	$0.579_{\pm0.008}$	$0.533_{\pm0.010}$	$0.672_{\pm0.009}$	$0.668_{\pm0.011}$	$0.672_{\pm0.009}$	$0.650_{\pm0.010}$
	3	$0.599_{\pm0.009}$	$0.592_{\pm0.010}$	$0.599_{\pm0.009}$	$0.561_{\pm0.010}$	$0.703_{\pm0.010}$	$0.706_{\pm0.011}$	$0.703_{\pm0.010}$	$0.686_{\pm0.011}$
	4	$0.617_{\pm0.009}$	$0.612_{\pm0.011}$	$0.617_{\pm0.009}$	$0.583_{\pm0.011}$	$0.720_{\pm0.010}$	$0.724_{\pm0.011}$	$0.720_{\pm0.010}$	$0.707_{\pm0.011}$
	5	$0.630_{\pm0.009}$	$0.628_{\pm0.011}$	$0.630_{\pm0.009}$	$0.601_{\pm0.010}$	$0.734_{\pm0.009}$	$0.739_{\pm0.011}$	$0.734_{\pm0.009}$	$0.723_{\pm0.010}$
	6	$0.643_{\pm0.009}$	$0.641_{\pm0.010}$	$0.643_{\pm0.009}$	$0.617_{\pm0.010}$	$0.745_{\pm0.009}$	$0.751_{\pm0.010}$	$0.745_{\pm0.009}$	$0.737_{\pm0.010}$
	7	$0.650_{\pm0.009}$	$0.649_{\pm0.010}$	$0.650_{\pm0.009}$	$0.625_{\pm0.010}$	$0.756_{\pm0.009}$	$0.764_{\pm0.010}$	$0.756_{\pm0.009}$	$0.749_{\pm0.009}$
	8	$0.659_{\pm0.009}$	$0.659_{\pm0.010}$	$0.659_{\pm0.009}$	$0.636_{\pm0.010}$	$0.766_{\pm0.009}$	$0.773_{\pm0.010}$	$0.766_{\pm0.009}$	$0.760_{\pm0.009}$
	9	$0.666_{\pm0.009}$	$0.666_{\pm0.010}$	$0.666_{\pm0.009}$	$0.645_{\pm0.010}$	$0.772_{\pm0.009}$	$0.783_{\pm0.009}$	$0.772_{\pm0.009}$	$0.767_{\pm0.010}$
	10	$0.673_{\pm0.009}$	$0.673_{\pm0.010}$	$0.673_{\pm0.009}$	$0.653_{\pm0.010}$	$0.776_{\pm0.009}$	$0.785_{\pm0.010}$	$0.776_{\pm0.009}$	$0.771_{\pm0.010}$

B Comparative Evaluation and Ablation of Pipeline Stages (10shots)

Table 3. Action-Level Results of Comparison Study and Ablation Study. The same training (10 shots, one new target behavior) and testing data were used to ensure consistency.

Methods	Acc	Prec	Rec	F1
**SVM**	0.809±0.006	0.864±0.004	0.809±0.006	0.778±0.008
**Random Forest**	0.779±0.006	0.832±0.006	0.779±0.006	0.738±0.008
**COVID-away**	0.740±0.005	0.812±0.004	0.740±0.005	0.698±0.006
**Itchtector**	0.745±0.006	0.808±0.006	0.745±0.006	0.696±0.008
**HandGesCus**	0.820±0.005	0.847±0.005	0.820±0.005	0.811±0.005
**SVM** (data augmentation & synthesis)	0.714±0.005	0.783±0.007	0.714±0.005	0.653±0.008
**Random Forest** (data augmentation & synthesis)	0.786±0.006	0.830±0.006	0.786±0.006	0.751±0.008
**Itchtector** (data augmentation & synthesis)	0.754±0.007	0.785±0.009	0.754±0.007	0.702±0.009
**WatchGuardian** w/o all	0.800±0.005	0.839±0.005	0.800±0.005	0.785±0.006
**WatchGuardian** with Stage 1 (pre-trained SSL model)	0.839±0.004	0.864±0.004	0.839±0.004	0.830±0.005
**WatchGuardian** with Stage 1 & 2 (finetuning)	0.841±0.004	0.872±0.004	0.841±0.004	0.829±0.005
**WatchGuardian** with Stage 1, 2, & 3 (data augmentation & synthesis)	**0.877±0.005**	**0.890±0.005**	**0.877±0.005**	**0.873±0.006**
